# Research Progress in Electroactive Polymers for Soft Robotics and Artificial Muscle Applications

**DOI:** 10.3390/polym17060746

**Published:** 2025-03-12

**Authors:** Yogesh Dewang, Vipin Sharma, Vijay Kumar Baliyan, Thiagarajan Soundappan, Yogesh Kumar Singla

**Affiliations:** 1 Department of Mechanical Engineering, Lakshmi Narain College of Technology, Bhopal 462021, India; yogeshd@lnct.ac.in; 2 Department of Mechanical Engineering, Medi-Caps University, Indore 453331, India; vipinsharma@medicaps.ac.in; 3 School of Sciences, Sanjeev Agarwal Global Education University, Bhopal 462022, India; vijay.b@sageuniversity.edu.in; 4 School of Science, Navajo Technical University, Crownpoint, NM 87313, USA; tsoundappan@navajotech.edu; 5 School of Engineering, Math & Technology, Navajo Technical University, Crownpoint, NM 87313, USA

**Keywords:** actuators, electro-active, polymers, force, strain, soft actuators

## Abstract

Soft robots, constructed from deformable materials, offer significant advantages over rigid robots by mimicking biological tissues and providing enhanced adaptability, safety, and functionality across various applications. Central to these robots are electroactive polymer (EAP) actuators, which allow large deformations in response to external stimuli. This review examines various EAP actuators, including dielectric elastomers, liquid crystal elastomers (LCEs), and ionic polymers, focusing on their potential as artificial muscles. EAPs, particularly ionic and electronic varieties, are noted for their high actuation strain, flexibility, lightweight nature, and energy efficiency, making them ideal for applications in mechatronics, robotics, and biomedical engineering. This review also highlights piezoelectric polymers like polyvinylidene fluoride (PVDF), known for their flexibility, biocompatibility, and ease of fabrication, contributing to tactile and pressure sensing in robotic systems. Additionally, conducting polymers, with their fast actuation speeds and high strain capabilities, are explored, alongside magnetic polymer composites (MPCs) with applications in biomedicine and electronics. The integration of machine learning (ML) and the Internet of Things (IoT) is transforming soft robotics, enhancing actuation, control, and design. Finally, the paper discusses future directions in soft robotics, focusing on self-healing composites, bio-inspired designs, sustainability, and the continued integration of IoT and ML for intelligent, adaptive, and responsive robotic systems.

## 1. Introduction

Soft robots are primarily made of deformable materials, which allow them to exhibit greater adaptability, resembling biological tissues and organs [1,2]. In contrast, robots made from rigid materials have limited ability to elastically deform or adapt to external constraints. Soft robots, however, offer enhanced capabilities by performing actions that are not feasible for conventional rigid robots. Furthermore, soft robots are safer to work with humans and are well-suited for handling critical tasks [3,4]. To achieve functionality similar to human beings, bio-inspired design and system integration in soft robots is a significant challenge. In the human body, only 15% of the mass is composed of rigid materials, with the remaining 85% made up of soft tissues [3,4,5,6]. It is well established that soft materials are excellent at dissipating energy from impacts, smoothing out discontinuities in force movements, and damping oscillations. Robots designed with soft materials are more adaptable and capable of natural fluid movements [5,6].

In soft robots, soft actuators play a crucial role, responding to various external stimuli and exhibiting large deformations [7,8,9]. A wide variety of soft actuators, such as hydrogels, shape memory alloys, shape memory polymers, carbon nanotubes, graphene, and EAPs, are commonly used in these robots [8,9,10,11,12]. EAP-based actuators eliminate the need for gears, bearings, and other components that add complexity, weight, and cost to conventional robots. Moreover, EAPs are flexible enough to be configured into desired shapes, and their properties can be engineered to adapt to external constraints [11,12,13]. EAP soft actuators have garnered significant attention due to their desirable characteristics, including affordability, ease of fabrication, flexibility, high actuation strain, light weight, high power density, mechanical compliance, structural simplicity, versatility, scalability, and the absence of acoustic noise [13,14,15,16].

EAP actuators exhibit large mechanical actuation when subjected to electrical stimulation, making them ideal for applications as soft actuators resembling human muscles. These unique capabilities also render EAP actuators highly suitable for a variety of fields, including mechatronics, robotics, automation, biomedical engineering, haptics, biotechnology, fluidics, optics, and acoustics [16,17,18,19,20,21].

The history of EAP actuators dates back to 1880, when a rubber strip, in the form of a cantilever beam with a point mass, exhibited elongation under the influence of an electric field. In recent years, EAP actuators have emerged as some of the most influential actuators in the development of soft robots [22,23].

Numerous studies have contributed to the advancement of EAP actuators, particularly for use in soft robots, with an emphasis on artificial muscle applications [24,25,26,27]. For example, Guin et al. [28] demonstrated that layered LCE actuators displaced a weight by nearly 0.5 mm, which was 2500 times heavier than the actuator itself. Shahinpoor [29] introduced a novel composite of monodomain nematic LCEs and conducting materials, which exhibited reversible strain exceeding 200% within seconds. In the realm of conducting polymer actuators, Madden [30] reported strains of up to 6%, strain rates of 4% s^−1^, forces up to 34 MN/m^2^, and power-to-mass ratios of 40 W/kg. Conducting polymer actuators were found to generate forces at least ten times greater than skeletal muscle in a given area, producing up to one thousand times more strain for a range of 1–10%.

Finkelmann and Shahinpoor [31] developed liquid crystal elastomer-graphite composites that displayed a volume expansion of nearly 53% when mixed with graphite powder. Davidson et al. [32] combined the inherent qualities of dielectrics and LCEs to enhance actuation speed, shape change programming, and conversion efficiency. Bar-Cohen and Zhang [33] noted that increasing the dielectric constant of polymer materials using support fillers can produce higher strains with low voltage requirements. Carpi et al. [34] explored the potential of EAPs for use as artificial muscle in biomimetic motion applications. Marin et al. [35] developed a new framework for finite element EAPs with multiple layers and large deformations. Kallitsis et al. [36] reviewed fluorinated EAPs and highlighted their suitability as insulating materials in organic electronic devices.

Xia et al. [37] found EAPs to be ideal for actuating various types of valves. Hartmann et al. [38] introduced a unique design for soft-focus tunable lenses, consisting of a liquid-filled elastomeric lens membrane inflated by EAPs to adjust focal length. Jo et al. [39] developed a new method to prepare polymeric electrodes on Nafion membranes, creating actuators with controllable pseudocapacitive layers. Kim et al. [40] emphasized the advantages of low-voltage iconic EAP actuators for applications, citing their preference over electronic EAP actuators due to their lower driving voltages (less than 3 V). Baughman et al. [41] concluded that conducting polymer actuators outperformed piezoelectric polymer actuators in terms of dimensional changes, work density per cycle, operating voltage, and electrically generated stresses.

The objective of this paper is to review the different categories of EAP actuators that mimic human muscle and can be used as soft actuators in the development of soft robots. Additionally, a comparison of these categories is outlined in terms of performance and operational parameters to guide the selection of the most suitable EAPs for specific applications.

## 2. Classification of EAPs

**Figure 1:**
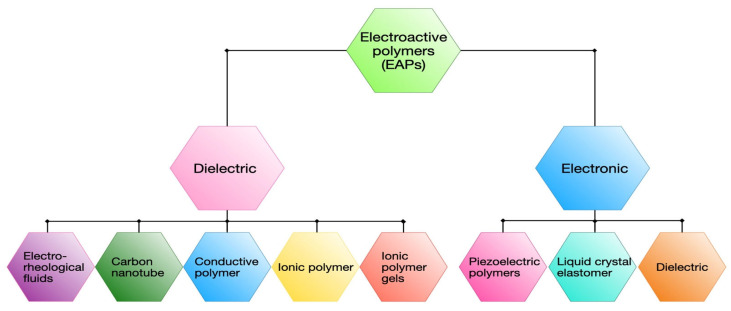
Types of electro-active polymers actuators.

EAPs can be broadly classified into two major categories: electronic EAP actuators (high voltage range) and ionic EAP actuators (low voltage range), as illustrated in Figure 1 [42,43]. Electronic EAP actuators operate through the driving force generated by an electric field, whereas ionic EAP actuators function via ion diffusion or mobility. Electronic EAP actuators can be further categorized into dielectric elastomers, LCEs, and piezoelectric polymers. Similarly, ionic EAP actuators are sub-classified into ionic polymer gels, ionic polymer-metal composites (IPMCs), conducting polymers, electrorheological fluids, and carbon nanotube-based actuators. Compared to ionic EAPs, electronic EAP actuators are more suitable for soft robotics applications due to their large strain capabilities, high energy density, and fast response time [25,44].

## 3. Dielectric Elastomers Actuators

**Figure 2:**
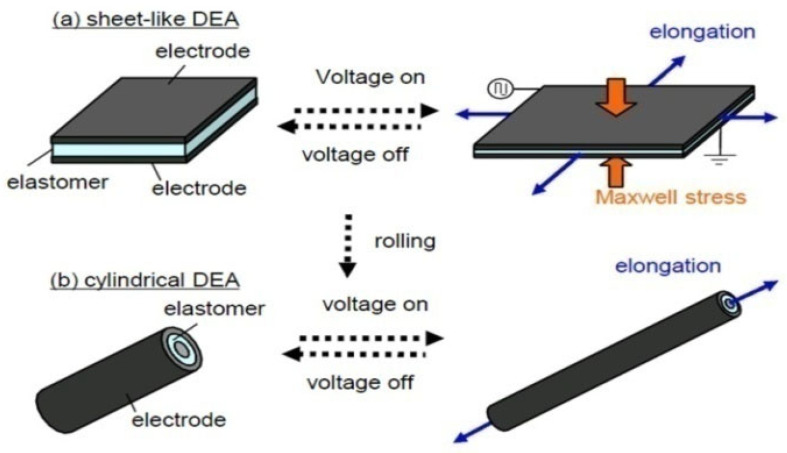
Schematic presentation of driving mechanisms of DEAs: (**a**) sheet-like DEA and (**b**) cylindrical DEA [53].

Dielectric elastomers are a class of EAPs that exhibit significant deformation when subjected to an external electric field [45,46]. Dielectric elastomer actuators (DEAs) are well-established as actuators, energy harvesters, and sensors. They are particularly prominent due to their large deformation capabilities, fast response times, light weight, low elastic modulus, high energy density, and low cost. The basic construction of a dielectric elastomer consists of a dielectric film sandwiched between two compliant electrodes, as illustrated in Figure 2 [47,48]. These actuators can be configured in various forms, including sheet-like DEAs and cylindrical configurations, as shown in Figure 2. Furthermore, DEAs can be adapted into different designs, such as planar, rolled, multi-layer stacked, folded, balloon, and diamond shapes, for a wide range of applications. Despite these varied configurations, the fundamental working mechanism and construction principles remain consistent across all dielectric elastomer actuators. DEAs are used in applications such as soft grippers, crawling robots, flying robots, humanoid robots, swimming robots, tunable lenses, and tactile displays [49,50].

The working mechanism of DEAs is straightforward, as depicted in Figure 2. When voltage is applied to the compliant electrodes, an electric field is generated within the dielectric film. The induced Maxwell stress due to this electric field causes the dielectric film to expand in area while contracting in thickness [45,46,47,48]. Since the dielectric film is the primary component, the material properties of the dielectric film are crucial for the actuator’s performance. To achieve optimal performance, the dielectric film must possess low viscosity, low modulus, a high dielectric constant and high electric breakdown strength [51,52]. Common materials used for dielectric films include acrylics, silicones, and polyurethanes. For the compliant electrodes, the required properties are high compliance, good stability, conductivity, and strong adhesion. Common electrode materials include carbon grease, carbon nanotube, carbon powder, and graphite [48,49,50].

**Figure 3:**
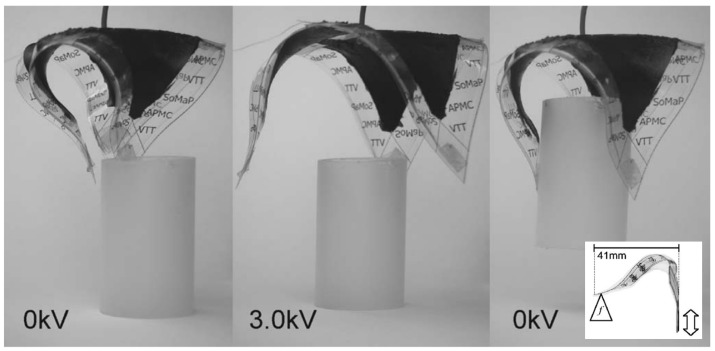
A tulip-shaped gripper based on dielectric polymers [56].

Soft grippers, designed to mimic human fingers, are commonly used for grasping objects. DEAs are an ideal choice for soft grippers due to their high flexibility and adaptability to various configurations [54,55,56]. In a study, Kofod et al. [56] developed a triangular or tulip-shaped gripper with three claws based on dielectric polymers. They demonstrated that when voltage is applied, the gripper opens to accommodate the object, and when the voltage is removed, the claws contract to securely hold the object, as shown in Figure 3.

## 4. Liquid Crystal Elastomers Actuators

Liquid crystal phases are unique molecular materials with large anisotropies in properties such as optical, dielectric, and mechanical anisotropies at the molecular level [57,58]. The significant shape anisotropy of liquid crystal molecules makes them especially attractive for actuator applications. However, liquid crystals themselves cannot exert or sustain mechanical stress due to their elastic properties, making pure liquid crystals unsuitable for practical actuator applications [59,60,61]. When liquid crystal molecules are chemically linked to form long chains, the flow of the material is obstructed, and it tends to become crystalline. If these chains remain flexible and are incorporated into liquid crystal structure, the resulting material, LCEs, retains some ability to flow [57,58,59,60,61]. LCEs are therefore well-suited for use in soft robotics actuators.

**Figure 4:**
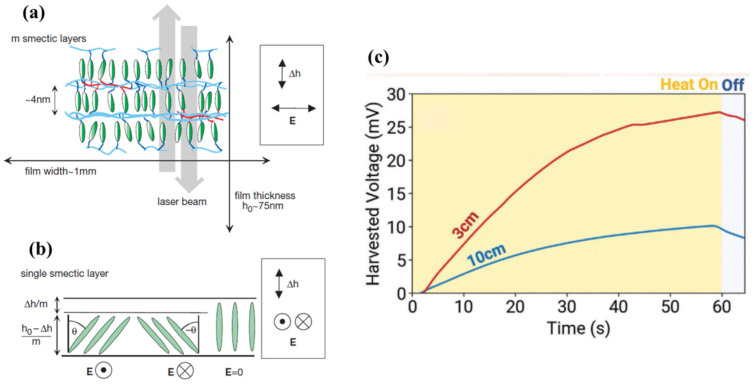
(**a**) The smectic layers are arranged parallel to the plane of the freely suspended film of thickness h_0_; (**b**) The electroclinic effect: the thickness of smectic layer decreases under an electric field; (**c**) Graph of energy harvesting from the front limb at the initial and final positions of the soft walker, highlighting the walker’s ability to move over to a power source and passively generate voltage during hibernation [62].

Figure 4a illustrates an LCE consisting of m (where m = 3) smectic layers of a certain thickness. A laser beam is used to measure the thickness of these layers. The diagram shows a single smectic layer both with and without an electric field applied. Under the electric field, the thickness of the smectic layer decreases because the molecules within the layer tilt at an angle θ, as depicted in Figure 4b. Additionally, LCEs have been integrated with soft, stretchable thermoelectrics, enabling them to be stimulated both thermally and electrically. This combination allows for thermal-to-electrical energy conversion and inherent active cooling capabilities. Figure 4c shows the harvested voltage from the front limb of a soft walker at both the initial and final positions. Notably, higher voltage output was observed when the front limb was positioned 3 cm from the heat source compared to when it was 10 cm away [62].

Table 1 outlines the various applied areas and potential applications of LCEs. These materials exhibit significant strain actuation even with a small increase in loading, though they also demonstrate low tensile strength and low stiffness [63]. Due to their strain actuation capabilities, Shahinpoor [29] observed that liquid crystals can contract by more than 200% in contrast to shape memory alloys, which are limited to a 4–5% contraction under load. Additionally, LCEs, with their uniform molecular orientation, can generate strains up to 400%, resembling muscle-like contractile forces [28]. On the other hand, linearly actuated LCE monoliths can achieve high strain rates, around 120% s^−1^, with a conversion efficiency of 20% [32]. The electrical conductivity of LCEs can be enhanced by blending metallic particles and conductive materials such as polypyrrole and carbon particles [29]. In another study on electrically driven LCEs, Fikelmann and Shahinpoor [31] found that the actuator’s performance was more dependent on the thermal heat conductivity of the network than on the material transfer process. When a low direct current voltage (0.5 to 5 volts) was applied, the LCE demonstrated a quick response time of approximately 4 s under a stress of 10 kPa, showing an average linear strain of 25% through contraction. The cooling time to return to its original length under a 10 kPa stretching load was around 4.4 s [31].

**Figure 5:**
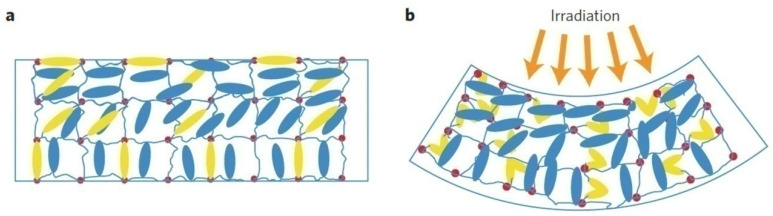
Schematic of bending induced by light radiation in a liquid crystal elastomer: (**a**) Mesogenic units (blue), crosslinks (red spots) and azo-crosslinker (yellow) in the trans state align parallel; (**b**) On exposure of light, the crosslinker undergoes trans–cis isomerization, contracting the network in the horizontal direction on top and dilating it on the bottom, causing a bend [64].

Guin et al. [28] noted that increasing the film thickness in LCE laminates did not change the material’s deformation behavior, but these laminates became capable of performing work on objects nearly 200 times heavier than the laminate itself. Davidson et al. [32] integrated the inherent capabilities of dielectric elastomers, such as anisotropic elasticity and Poisson’s ratio, with the patterning of thin LCE films to create a fast, highly efficient actuator with shape-programming capabilities. These dielectric LCE actuators, made from rubber polymers with anisotropic bulk properties, can exert loads over 700 times their own weight. LCEs consist of a weakly crosslinked polymer network (Figure 5a) with mesogenic side chains that have a nematic order [28]. When exposed to light, the crosslinker undergoes trans-to-cis isomerization, causing the entire LCE film to bend in a specific direction (Figure 5b). After the energy is removed, the LCE returns to its initial state.

## 5. Ionic Polymers

### 5.1. Ionic Polymer Metal Composites

**Figure 6:**
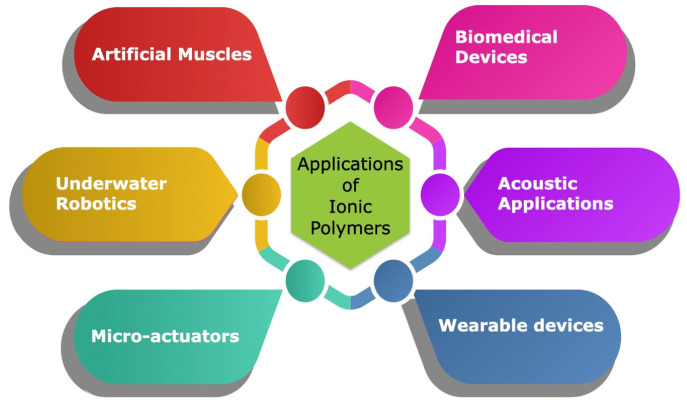
Applications of ionic polymers.

Ionic polymer metal composite (IPMC) actuators have emerged as an alternative, cost-effective solution to conventional polymer-based actuators. Nesser and Wu [65] highlighted that the electro-mechanical actuation behavior of IPMCs depends on factors such as the structure of the backbone ionic polymer, the conductivity of the metal electrodes, the morphology, the nature of the cations, and the level of hydration. They found that electrode morphology, the ionomer, the counter ion, and the degree of hydration are key determinants in the electromechanical response of the IPMC actuator. Figure 6 illustrates various applications of ionic polymers.

It has been observed that the interfacial area between the electrode and the polymer electrode in IPMC actuators is a crucial factor that significantly influences both the actuation response and electrochemical behavior. In a study by Noh et al. [66], Nafion metal composite actuators were prepared using a replication technique. The results showed enhanced bending performance, with the magnitude of the IPMC increasing by about 50% at 3 V due to the introduction of a larger interfacial area.

**Figure 7:**
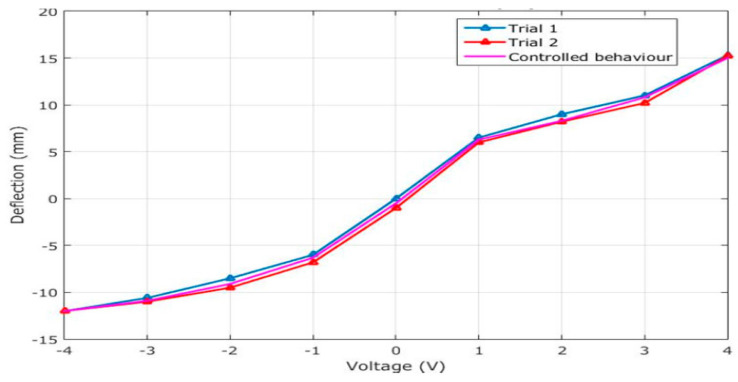
Deflection behavior of the SPVC-PTA-Pt polymer actuator obtained experimentally [68].

IPMC actuators not only utilize charges but also store them within the material. Studies have shown that samples with low surface resistivity generate larger blocking forces. In Nafion IPMC actuators, the current response to applied voltage is often insignificant [67]. Kim and Shahinpoor [67] suggested that the practical application of IPMC actuators, in terms of market viability, depends on generating higher force densities while maintaining optimal manufacturing costs. An analysis of the deflection behavior of sulfonated polyvinyl chloride (SPVC) and phosphotungstic acid (PTA) IPMCs with respect to input voltage showed that increased voltage resulted in larger deflections. Figure 7 illustrates the influence of input voltage on the bidirectional deformation behavior of the actuator. In several trials, a gap was observed in the SPVC-PTA-Pt polymer actuator, creating hysteresis in the bidirectional deflection behavior. However, the tip deflection increased with increasing input voltage, and errors in the analysis were minimized by applying control systems. Stabilized bending behavior was achieved through repeated voltage cycling [68].

There are various types of IPMCs that can function as actuators. One such class is aqueous electrolyte-based IPMC actuators, which exhibit rapid response due to their good ionic conductivity [69]. However, the performance of these actuators is limited by issues such as electrolyte evaporation and water leakage. Ionic liquids, while having lower ionic conductivity compared to aqueous electrolytes due to higher viscosity, offer benefits such as non-volatility and larger ion radius. These properties allow ionic liquid-based IPMC actuators to generate higher blocking forces and bending amplitudes [40,69]. Actuation speed in IPMC actuators is influenced by the capacity and electrical conductivity of the electrodes, as well as the ionic conductivity of the polymer membrane [69]. High performance in IPMC actuators can be achieved by regulating the charge distribution in polymer electrolytes, which improves electromechanical response and speeds up ion transport. The cations and the cation/anion interactions play a significant role in the actuation mechanisms of ionic liquid polymer actuators [40]. The applications of IPMC actuators are summarized in Table 2.

**Figure 8:**
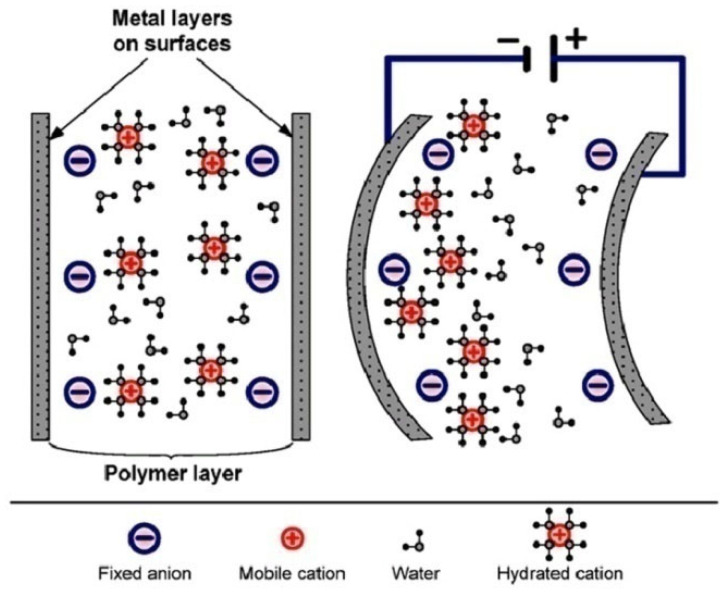
Schematic of the operating principle of an IPMC actuator [70].

When a low voltage is applied to the electrodes, the transport of hydrated cations and electrostatic interactions within the IPMC sheet cause it to bend, allowing the IPMC to function as an actuator, as shown schematically in Figure 8.

### 5.2. Ionic Polymer PVC Gels

Ionic polymer gels represent a promising category of EAPs actuators. These gels are capable of larger deformations and exhibit high actuation speeds, even under low driving voltages. Additionally, they demonstrate durable, stable performance with significant electrically induced bending deformation [71,72]. Li et al. [71] developed an ionic polymer gel actuator that utilizes flexible electrodes, allowing both thickness and area deformations in the gel. This planar polyvinyl chloride (PVC) gel actuator can stretch up to 600%, and it showed superior flexibility, lighter weight, and a more compact size compared to multilayered PVC gel actuators.

In the development of plasticized PVC gel actuators, Xia et al. [72] identified the importance of the distance between the electrodes and the deformation of the gel, as these factors significantly impact performance due to the gel’s very soft nature. They also found that the dibutyl adipate (DBA) had a pronounced effect on the space charge density, noting that optimizing DBA content was crucial for achieving a higher polarization response.

In a study by Basinki and Christain [73], it was discovered that thermally activated shape memory alloys, such as indium-titanium alloys, exhibited similar behavior by returning to their original state after deformation when subjected to higher temperatures. Li et al. [74] developed a planar PVC gel actuator that displayed a maximum strain of 21% and a maximum output stress of 600 kPa at a low voltage of 120 V (15 V/μm). This performance is comparable to the levels of skeletal muscle, making it suitable for use in soft robotics. It was further reported that the deformation of PVC gel actuators is primarily caused by the force between two electrodes, resulting from the polarization of accumulated charge density [70].

**Figure 9:**
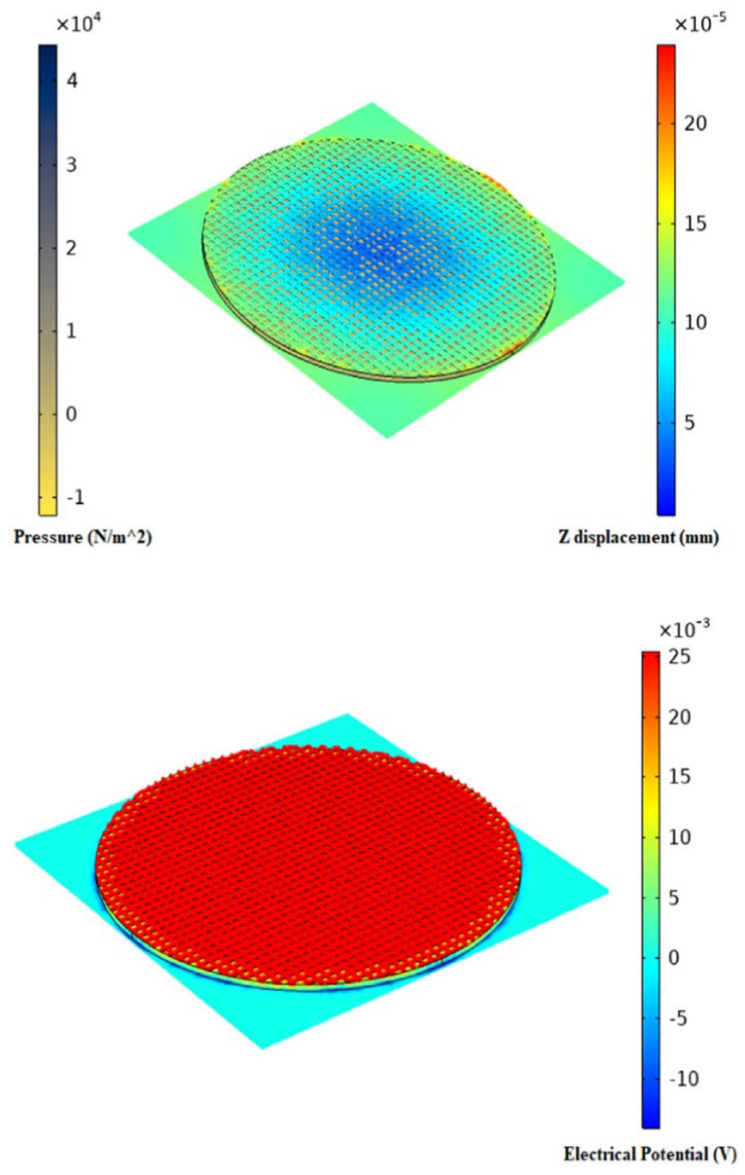
Contour plots of mechanical pressure due to the input force to the anode, mechanical deflection, and electric potential in response to mechanical deflection due to input force through FEM simulation [75].

In an attempt to investigate the performance of ionic polymer PVC gel as a compression sensor, finite element simulation was conducted to study its response to mechanical force using the COMSOL software package. It was observed that PVC gel models deflected as mechanical pressure was applied across the entire model. Figure 9 illustrates the contour plot of pressure displacement in the z-direction and the electric potential. The results clearly show that applying a mechanical compressive load to the PVC gel caused mechanical deflection, which subsequently generated an electric potential in response to the applied force [75].

## 6. Piezoelectric Polymers

**Figure 10:**
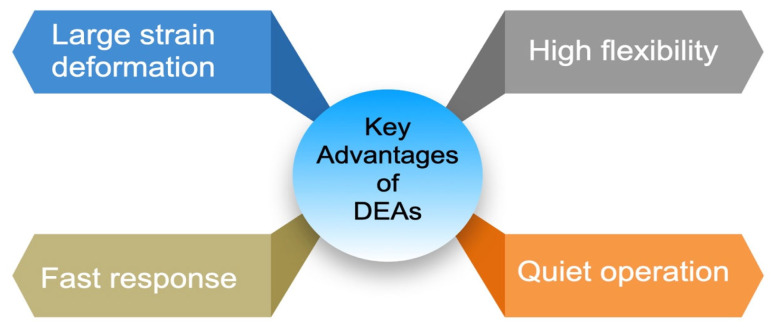
Key advantages of piezoelectric polymers.

Piezoelectric materials are characterized by their ability to generate electrical charges when subjected to mechanical stress. They also exhibit the converse piezoelectric effect, where mechanical deformation or output occurs in response to an applied electrical field [76,77]. Figure 10 highlights the key advantages of piezoelectric polymers.

**Figure 11:**
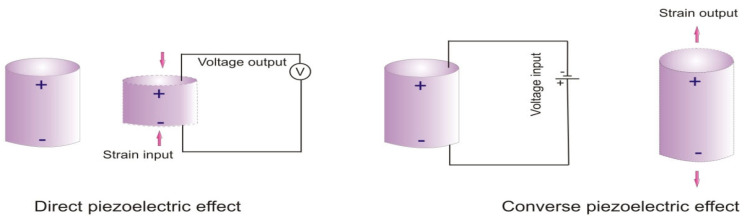
Schematic presentation of direct piezoelectric and converse piezoelectric effect.

A schematic representation of the direct and converse piezoelectric effects is shown in Figure 11. While various ceramic materials demonstrate piezoelectricity, certain polymers, such as PVDF and its copolymers, polyhydroxybutyrate, and polylactic acid, also exhibited piezoelectric properties. PVDF, in particular, shows strong piezoelectric behavior due to the high electronegativity of fluorine atoms compared to carbon atoms [78,79]. Piezoelectric polymers offer several advantages over ceramic piezoelectric materials, including higher flexibility, higher electric breakdown fields, non-toxicity, greater elastic energy density, and lighter weight [80]. PVDF specifically stands out due to its low permittivity, flexibility, ease of fabrication, light weight, wide frequency response, cost-effectiveness, and biocompatibility. These attributes make PVDF suitable for a wide range of applications, including tactile sensors, pressure sensors, stress sensors, generators, vibration transducers, and accelerometers [79].

Kim et al. [81] developed a sensor-based robotic micro-gripper utilizing piezoelectric PVDF film sensors. They employed superelastic NiTi alloy for actuation within the micro-gripper. The PVDF film and superelastic alloy work together to enable precise force sensing, enhancing the grip displacement. The study found that PVDF demonstrated superior properties in force feedback and exhibited a high signal-to-noise ratio, allowing the NiTi alloy micro-gripper to detect gripping forces with remarkable sensitivity. Tian [82] designed a robotic identification system using PVDF soft sensors to discriminate different materials. The experiment result showed that the system was approximately 95% effective and reliable in distinguishing materials based on their textures. Materials with different coefficients of friction generate unique signal inputs to the soft sensors, making material differentiation straightforward.

To replicate the behavior of human skin, flexible tactile sensors based on arrays of piezoelectric transducers were developed by Seminara et al. [83]. They found that ink jet printing of PVDF film was a suitable method for creating large-area artificial skin. Kimoto et al. [84] developed a PVDF-based tactile sensor for identifying various materials. The sensor measures the electrical properties and contact voltage generated by electrostatic and piezoelectric effects between the material and the sensor. Material discrimination was achieved through contact voltage, which varies with the hardness and viscosity of the material. Hosoda [85] fabricated a fingertip prototype made of metal and soft material, with receptors randomly embedded to mimic human skin and bone. Metal was used to simulate bone, while silicon represented skin. Strain gauges and PVDF films were embedded in the soft material and silicon skin to serve as the receptors.

Namvarrechi et al. [86] developed a sensor for endoscopic grasper applications by depositing poly vinyledene difluoride-trifluoroethylene (PVDF-TrFE) between aluminum electrodes. They used post-annealing treatments to increase the electroactive β-phase content in PVDF-TrFE, enhancing the material’s piezoelectric properties.

Gupta et al. [87] fabricated electronic skin for humanoid robots by using a sensing material composed of PVDF-TrFE and Barium Titanate (BaTiO_3_) nanoparticles. The polymer film ensured the sensor’s flexibility, while the BaTiO_3_ nanoparticles provided high sensitivity to touch and temperature. This sensor was capable of detecting both touch and temperature due to the addition of BaTiO_3_.

**Figure 12:**
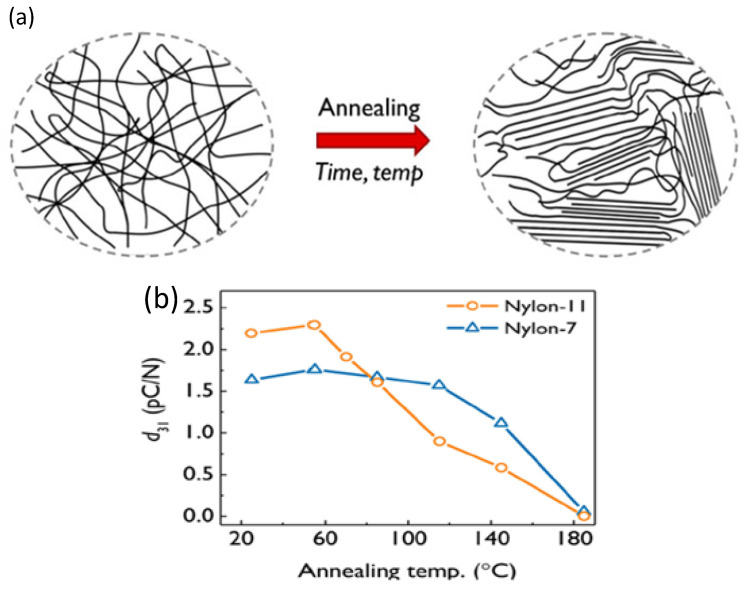
(**a**) A schematic representation of polymer crystallization as a result of annealing; (**b**) The influence of annealing temperature on the piezoelectric properties of Nylon-11 and Nylon-7 [88].

In one analysis of piezoelectric materials, annealing was found to improve the crystallinity of the polymers and enhance their material properties. Figure 12a illustrates the effect of annealing on piezoelectric polymers. It is important to note that while annealing promotes crystallization, the polymer remains isotropic, and the crystallization alone is insufficient to eliminate the center of symmetry. Figure 12b shows that annealing increases the ordering of polymer chains, restricting dipole rotation. This results in reduction of the ferroelectric and piezoelectric properties of the polymers, leading to a decrease in the piezoelectric coefficient for two different types of nylon. Additionally, the piezoelectric mechanisms were found to differ between two nylon variants: Nylon (1-1) and Nylon-7 [88].

## 7. Conducting Polymers

Conducting polymers are a distinct category of EAPs that involve ion transport and typically require an ion reservoir for actuation. These materials are electronically conductive, and their oxidation/reduction reactions within an electrolyte lead to volumetric strain [69,89]. A key factor influencing strain generation in conducting polymer actuators is the transfer of charges between the anode and cathode. This charge transfer process is not dependent on the applied voltage or the electrical field [41,90]. In this mechanism, the electrochemical oxidation state involves the addition or removal of charges from the polymer backbone, while charge balance is maintained by the movement of ions. The use of ionic liquid electrolytes in conducting polymers has been shown to significantly extend the cycle life, by nearly 1 million cycles to several tens of thousands of cycles [63]. Conducting polymer actuators typically consist of monomers such as pyrrole, aniline, and thiophene. They are also referred to as conjugated polymer actuators, which exhibit high actuation speeds. However, these actuators require contact with organic solvents to achieve high strain capabilities. Examples of conjugated polymers used for actuator manufacturing include polyepyrrole, polyaniline, polyethylenedioxythiophene, and polystyrenesulfonate [40,91]. In addition to their speed, conducting polymer actuators can generate forces up to ten times greater than skeletal muscle for a given cross-sectional area and can produce forces up to 1000 times greater for strain ranges of 1–10% [30]. The reduced weight and volume of conducting polymers, resulting from the limited amount of electrolyte required for ionic conductivity, are notable advantages. Another benefit is their ability to operate at lower voltage ranges, making them suitable for medical actuator applications [41,92,93]. However, a limitation of conducting polymer actuators is their slow response times, resulting in a lower power-to-mass ratio compared to other actuator technologies [30]. Table 3 outlines the potential applications and areas where conducting polymer actuators are being utilized.

**Figure 13:**
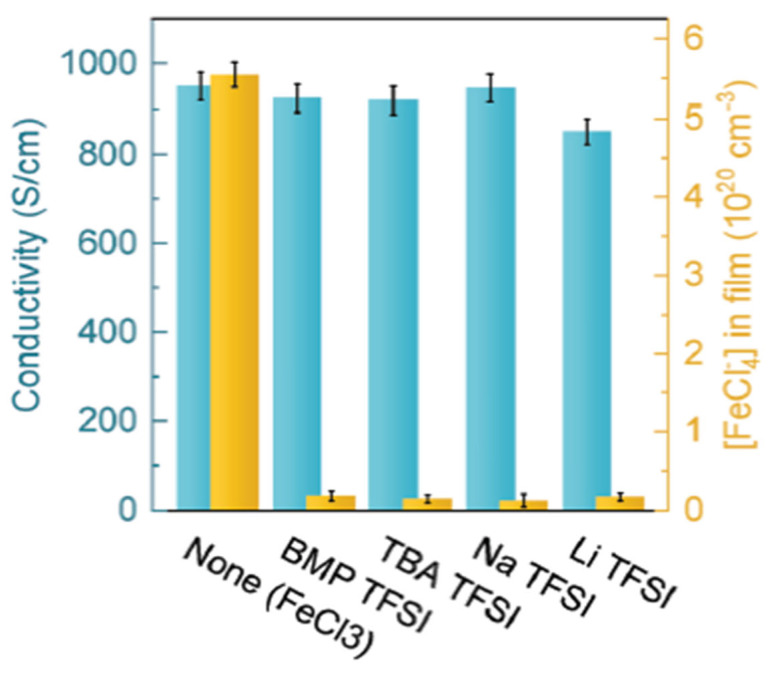
Bar graph showing conductivity (blue, left axis) and extracted FeCl_4_^−^ (yellow, right axis) for films [45].

The concept of molecular doping in conducting polymers has gained significant popularity in recent years. This category of conducting polymers finds applications in bio-electronics and thermo-electrics [94,95,96]. Figure 13 presents a bar graph chart comparing the ion exchange efficiency of conducting polymers cations. Notably, apart from Lithium Bis(trifluoromethanesulfonyl)imide (Li TFSI), all other conducting polymers demonstrated high ion exchange efficiency. Li TSFI, however, exhibited a lower doping level (as indicated by the extracted FeCl_4_^−^ concentration) and lower electrical conductivity compared to other conducting polymers cations, such as FeCl_3_, BMPTFSI, TBA TFSI, and Na TFSI. This reduction in conductivity and doping level was attributed to the higher content in Li TFSI, which led to a decrease in the reduction potential of Fe^3+^. It was also observed that all cations achieved electrical conductivity greater than 600 S cm^−1^ [97].

In recent years, researchers have focused on the development of conducting polymers for use in soft actuators, particularly for soft robotics applications. Ma et al. [98] integrated actuation and strain-sensing functions in conductive MXene-encapsulated liquid metallic hydrogels. These hydrogels serve as bio-inspired, self-sensing, soft actuators, detecting movement by monitoring changes in resistance. To further enhance the autonomy of conducting polymers, Hu et al. [99] introduced a soft actuator made from a conductive polymer ionic gel. This actuator serves dual purposes, functioning both as a thermal sensing actuator and as a sensor for radiation emitted by the human body, enabling more intelligent decisions during human interaction. In another advancement, Liu et al. [100] introduced dopamine-decorated polypyrrole nanofibers embedded in polyethylene glycol diacrylate to form a bilayer hydrogel. This structure demonstrated excellent electrical conductivity, self-sensing ability, thermal sensing capabilities, and underwater grasping abilities.

In the field of artificial muscle development based on conducting polymers, Hu et al. [101] developed coiled conductive polymer yarns as a lightweight and cost-effective alternative to carbon nanotubes for artificial muscles. These coiled yarns are capable of producing exceptional contractile strain, exceeding 11% at high stress levels of 5 MPa, and can lift loads over 4000 times the weight of the artificial muscle itself. These findings highlight conducting polymers as a prime material for the development of soft actuators in soft robotic applications.

## 8. Magnetic Polymer Composite

MPCs are materials that combine magnetic particles with a polymer matrix to create material with magnetic properties [102]. The unique properties of magnetic particles, combined with the flexibility of polymer matrices, offer significant advantages for advanced material design. MPCs typically contain iron oxides or iron-oxide-based materials, which are stable and easy to prepare [103,104,105,106,107,108]. These composites have garnered considerable attention in various fields, including biomedicine, electronics, and environmental engineering, due to their tunable magnetic, mechanical, and thermal properties [109]. MPCs are hybrid materials that combine magnetic nanoparticles (MNPs), which exhibit such excellent features as small size, high surface area, and active surfaces. These properties can be successfully modified for low toxicity and superparamagnetism [110]. The synthesis of MPCs involves incorporating magnetic particles into a polymer matrix. The uniform dispersion of MNPs plays a crucial role in determining the final properties of the composite. Various methods have been developed for preparing MPCs, some of which are discussed below.

**Figure 14:**
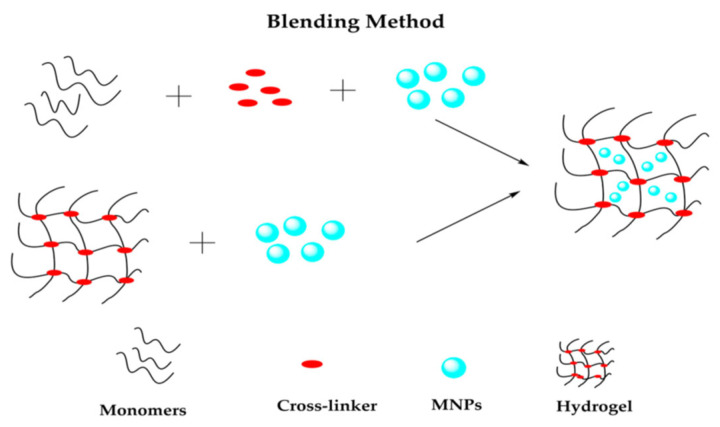
Representation of blending method to prepare magnetic hydrogel [117].

**Blending method:** The blending method is one of the most straightforward techniques for mixing organic-inorganic nanocomposites. In this process, the host polymer and nanoparticles are combined through melt and solution blending [111]. This method promotes a homogenous mixture of the polymer and magnetic particles, which is essential for nanocomposite materials. Melt blending is flexible, cost-effective, and environmentally friendly. Research has examined the effects of process parameters and various polymer composites on blending performance. For example, Chung et al. [112] used blending to synthesize cross-linked shape memory polyurethane/iron oxide magnetic composites and observed good mechanical and shape-memory qualities. Similarly, the melt blending of low-density polyethylene, polybenzoxine, and cobalt ferrite enhanced magnetic properties while maintaining the material’s structural flexibility [113]. However, at high filler concentrations, the melt-blending process can lead to poor filler dispersion, resulting in agglomeration and intercalation [114]. Despite this, the main advantage of this process is the ability to produce large quantities of MPCs through extrusion. Nonetheless, this method has certain limitations. Another commonly used blending approach for MPCs is solution-blending or solvent-casting [115,116]. In this method, magnetic hydrogels, which involve the physical encapsulation of MNPs into the hydrogel matrix, can be prepared, as shown in Figure 14 [117].

**In situ polymerization:** In situ polymerization is a method in which monomers are polymerized in the presence of fillers or additives, allowing the creation of composites with well-dispersed fillers. This method bypasses the strict thermodynamic constraints typically associated with the polymer intercalation process, making it more effective for producing MPCs with homogeneous filler distribution [118,119]. The dispersion of inorganic nanoparticles within the monomer and the stepwise polymerization process significantly influence the final properties of the composite.

Several synthesis techniques have been explored, including dispersion, suspension cross-linking, and inverse emulsion polymerization. One example of an in situ polymerization approach is the development of a superparamagnetic core-shell nanocomposite using poly (m-aminobenzenesulfonic acid) and iron oxide (Fe_2_O_3_, Fe_3_O_4_) particles. This method resulted in an MPC with enhanced power-conversion performance and a saturation magnetization value of approximately 40 emu/g [120].

**Figure 15:**
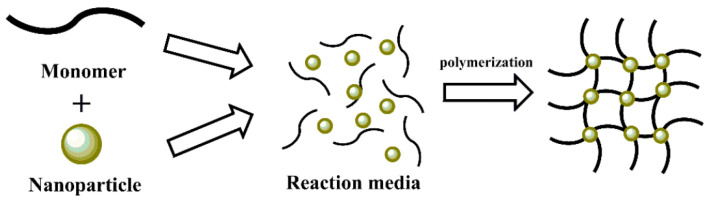
Schematic representation of in situ polymerization [122].

A similar method has been applied to composite hydrogels, where polypyrrole and iron oxide nanoparticles were prepared and incorporated into a polyvinyl alcohol matrix [121]. The resulting hybrid MPC demonstrated excellent mechanical, electrical, and magnetic properties, making it a promising candidate for use in biomedical applications as an electronic device. Figure 15 illustrates the general process of MPC formation by in situ polymerization [122].

**Molding:** Molding is a technique used to replicate patterns through soft lithography. In this process, the temperature of the mold wall exceeds the melting point of the polymer, facilitating the more rapid flow of material into the cavities. MPCs are prepared via molding by combining magnetic fillers with polymeric precursors, then curing the mixture to form specified shapes or structures [123]. Various molding techniques, such as injection molding and resin transfer molding, involve filling a mold under pressure and heat.

**Figure 16:**
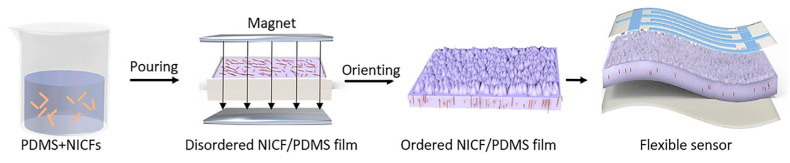
Tactile sensor based on magnetically aligned nickel-coated carbon fibers (NICFs) in PDMS, as well as the fabrication process and resulting sensor [125].

Recently, polydimethylsiloxane (PDMS) micropatterning has been employed to coat Fe_3_O_4_ nanoparticles with carbon nanotubes mixed with cross-linked poly(cyclotriphosphazene-co-4,4′-sulfonyldiphenol). This composite serves as a photothermal magnetic filler [124]. Jiang et al. [125] successfully created a flexible pressure sensor by incorporating nickel-coated carbon fibers into PDMS and aligning them with an external magnetic field during the curing process, as shown in Figure 16. This method resulted in a significant increase in the sensor’s sensitivity by more than two orders of magnitude.

Other very well-known methods, such as coprecipitation, chemical vapor deposition, and grafting, have also been used to prepare MPCs. Recently, nanoscale MPCs have generated significant interest due to their diverse applications. These include for electronic devices, sensors and transducers, magnetic storage, electromagnetic and microwave absorption, and magnetic actuators [125,126].

In the early 2000s, MPCs were produced by incorporating micrometer-sized iron carbide particles into elastomer matrices, resulting in an increased elastic modulus under the influence of an external magnetic field [127]. MPCs also show promise for micro/nanoplastics separation and degradation. Urso et al. reported that γ-Fe_2_O_3_/Pt/TiO_2_ microrobots could capture 97% of PS-COOH nanoparticles (50 nm) thanks to their stronger electrostatic interactions and the multilayered stack morphology exhibited in their study. This performance outpaced the capture rate of 50% achieved by MXene microparticles [128].

MPCs are also valuable for electromagnetic interference shielding. By leveraging the magnetic properties of the particles, they effectively absorb and attenuate electromagnetic waves, providing protection against unwanted electrical signals [129]. In soft robotics, MPCs serve as flexible actuators that respond to external magnetic fields, enabling controlled movement and deformation [130]. In biomedical applications, MPCs are used for magnetic resonance imaging contrast enhancement and targeted drug delivery. Additionally, they are employed in magnetic hyperthermia, a cancer therapy method that utilizes the heating properties of MNPs in an alternating magnetic field to selectively eliminate cancer cells while preserving healthy tissues [131]. These composites are also utilized in sensors and energy harvesting devices, converting magnetic energy into electrical signals or mechanical motion [132].

## 9. Recent Developments in Soft Robotics

**Figure 17:**
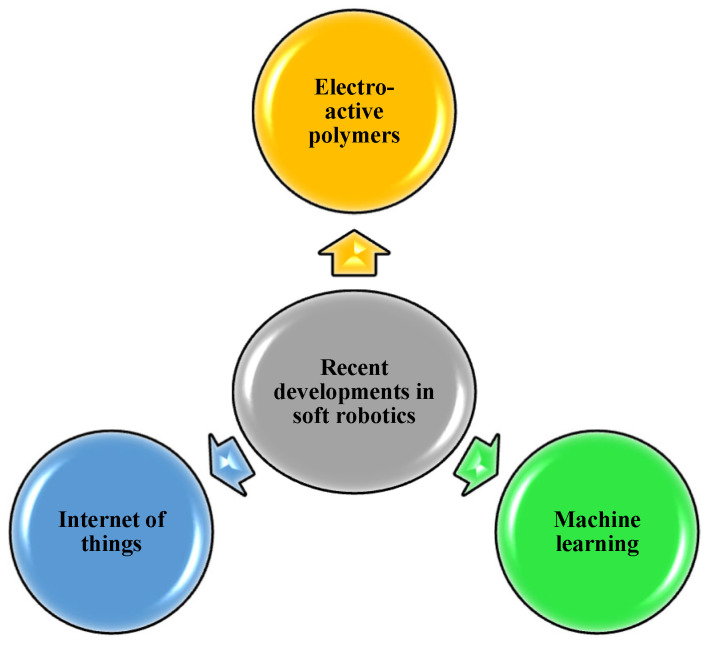
Recent developments in soft robotics.

Recent developments in robotics have identified the IoT, ML, and EAPs as the key drivers of innovation, as illustrated in Figure 17.

### 9.1. Electro-Active Polymers

EAPs have emerged as a major contributor to developments in soft robotics. Lu et al. [133] identified CoNC-700, a surface material known for its superior electromechanical performance, with the largest specific capacitance, as a significant advancement.

Khalid et al. [134] explored the use of magneto-active smart materials in 3D printing for advanced actuators in soft robotics, finding that these materials enable the creation of soft magnetic structures that improved deformation through magneto-thermal coupling actuation. Hassan et al. [135] reviewed electro active shape memory alloys and highlighted the progress in material engineering strategies for shape memory enhancement, leading to the development of electro-active shape memory polymer composites. These composites, with their excellent properties and ease of control, are now used in biomedicine, electronics, and robotics. Yang and Wang’s [136] work on high-performance electroactive and magnetostrictive materials significantly advanced soft robot actuation by enabling efficient motion and deformation due to their exceptional mechanical properties, rapid reaction time, and high energy conversion efficiency.

Reghunadhan et al. [137] investigated the use of electroactive materials, such as carbon nanotubes and polymers, in the production of sensors and actuators. Their lightweight, flexible, and durable properties make them ideal for robotic applications. Trumpler et al. [138] developed a 3D-printed ionic polymer actuator that demonstrated high bending and blocked forced features at a low cost. This biocompatible ionic polymer membrane actuator is ideal for delicate and sensitive systems, owing to its one-shot manufacturing process. Jumet et al. [139] suggested that future research could involve larger population sizes, more inclusive evaluation thresholds, ML or text-based techniques, and deeper content analyses to further enhance understanding in this field.

Chen et al. [140] highlighted the potential of EAPs for advanced haptic performance, enabling sophisticated, customized interactions through artificial intelligence (AI) integration, which enhances their application potential. Bernat et al. [141] emphasized the benefits of dielectric electroactive polymers, including high strain values, large actuation forces, quick reaction times, and great flexibility. Biswal [142] further pointed out that EAPs are ideal for soft robotic actuators since they do not require integration into rigid components for operation. Fernandes et al. [143] stressed the importance of careful selection when producing ionic liquid-based materials, as the interaction between the ionic liquid and polymer matrix plays a crucial role in enhancing functional response. Theoretical simulations on IL-polymer and IL-solvent interactions are necessary for a deeper understanding of these behaviors.

Kanaan et al. [144] highlighted the early-stage development of EAPs and additive manufacturing processing of EAPs. To address rheological challenges, researchers have blended non-conductive materials with metallic particles or constructed multilayered structures to induce form morphing following electrical stimulation. Current research focuses on improving the processability of materials and their impact on electroactive properties, tackling the challenges associated with traditional EAP production.

Jo et al.’s [145] study emphasized the versatility of EAP actuators in fields like soft robotics, biomimetics, wearable devices, and haptic technologies, thanks to their flexibility, lightweight nature, and simple fabrication process. Deng and Li [146] advocated for comprehensive research in biomimetics, stressing the importance of imitating biological systems like the sol–gel transitions, rather than focusing only on form replication. Enyan et al. [14] discussed the future of smart material-based soft actuators in adaptive systems, focusing on organisms’ ability to respond to environmental stimuli and exhibit complex motions.

Beregoi et al. [147] developed a bioinspired fibrillary artificial muscle with both intrinsic sensing and actuation capabilities. Their research revealed that mechanical sensing qualities decreased as the applied load increased, with certain electrochemical parameters becoming load-dependent until saturation occurred, such as 21.1 mg. Bruns et al. [148] explored PEDOT/Polypyrrole core-sheath fibers for conducting polymer artificial muscles, examining their tensile stability in both dry and wet conditions. These fibers are particularly well-suited for actuating in aqueous electrolyte systems, making them intriguing for soft robotics and wearable actuators, particularly in textile applications.

In addition to recent advancements in EAPs, it is important to note that the selection of EAPs for specific applications is critical in the context of soft robotics. The choice of EAP largely depends on the intended application and environmental conditions. Beyond the application itself, key factors such as operating voltage, response time, actuation strain, and efficiency are essential to consider when selecting the most suitable EAP. A comparison of different EAPs based on these major parameters, including key highlights and challenges, is provided in Table 4.

From Table 4, it is evident that for high actuation strain, DEAs, liquid crystal elastomers, and ionic EAPs are the preferred options, as they exhibit significant actuation strains. DEAs, due to their high actuation strain, are commonly applied in artificial muscles, biomimetic actuators, and haptic feedback systems. In applications requiring fast response and high sensitivity, piezoelectric polymers are favored, although they require high voltage and exhibit relatively small actuation strains (0.1–1%). These polymers serve well in both sensor and actuator roles, and are used in sensors, wearable electronics, and energy harvesting applications.

For applications with low power requirements, ionic EAPs are a viable choice, though they require constant hydration. Ionic EAPs are widely used in biomedical devices, artificial muscles, micro-actuators, underwater robotics, and artificial fins. In electronics, conducting polymers are utilized for their electrical conductivity; however, they are characterized by slower response times and degradation over repeated cycles.

In summary, recent advances in soft robotics emphasize the importance of IoT, ML, and Electroactive Polymers (EAPs). EAPs, including materials like CoNC-700, magneto-active smart materials, and electro-active shape memory alloys, have been improved in actuation, deformation, and material properties. Research is also focused on actuators and sensors made from carbon nanotubes, ionic polymers, and conducting polymers, as they offer flexibility and high performance. The choice of EAPs for soft robotics depends on factors such as voltage, response time, actuation strain, and efficiency. Dielectric elastomers and ionic EAPs are preferred for high actuation strains, while piezoelectric polymers are used for fast response and high sensitivity. Ionic EAPs suit low-power applications but require constant hydration, and conducting polymers are useful in electronics but degrade over time. EAPs show significant promise in biomimetics, wearable devices, and adaptive systems, with broad applications in soft robotics and beyond.

### 9.2. Machine Learning

Machine learning presents a transformative opportunity to elevate fields like soft robotics to new heights. Yin’s [155] study on the machine-learning-accelerated design of structural components for deep-sea soft robots demonstrated that an ML-based algorithm can reduce design time by seven orders of magnitude compared to traditional finite element method (FEM)-based approaches. This significant reduction accelerates the design of miniature pressure vessels with high precision and efficiency. Chin et al. [156] explored the potential of ML for soft robotic sensing and control, showcasing the promising results of both supervised and reinforcement learning in simulation and real robotic systems. Their work highlighted how data-driven, empirical approaches can effectively address challenges in soft robotics. Yao et al. [157] further advanced this field by demonstrating that magnetic soft robots can autonomously move without human guidance using deep reinforcement learning. This approach enables the robots to adapt intelligently to various magnetization patterns and magnetic field constraints. Akurda et al. [158] focused on deep learning techniques in soft robotics and identified key areas for future development, including generative AI, deep supervised learning, deep reinforcement learning (DRL), and the generalization of deep learning models. These technologies are expected to drive further advancements in soft robotic capabilities.

Yasa’s [159] research introduced the Koopman operator as a tool for modeling soft robotic dynamics through dynamic mode decomposition. This method linearizes the nonlinear system dynamics in a high-dimensional latent state space, making it possible to apply linear control methods. This approach has been extended to manage stochastic system dynamics, enhancing its utility in soft robotics. Terrile et al. [160] developed a multilayer perceptron neural network model capable of approximating nonlinear functions. However, achieving an acceptable margin of error requires a significant amount of data. To address this, they proposed a system that efficiently extracts data from finite element simulations, enabling more effective modeling of direct kinematics through ML techniques. Tsompanas et al. [161] leveraged ML to improve the efficiency of microbial fuel cells (MFCs) as energy sources for soft robotics. By utilizing the NARX model, which supports both open-loop and closed-loop modes, they enhanced training accuracy and adaptability during various application phases. This advancement significantly boosts the use of MFCs as reliable energy sources in soft robotic systems. Raeisinezhad et al. [162] employed model-based optimization and DRL for the design optimization of soft robotic actuators. They found that DRL is pivotal in designing actuators that effectively decouple horizontal and vertical motions, ensuring the necessary displacement for their intended applications.

Johnson et al. [163] utilized error-driven deep neural networks to train soft robots, focusing on an analytical model based on physics. Their approach is smaller, faster, and requires less training data compared to fully model-free learning strategies, as it only needs to learn minor adjustments. This is particularly beneficial when collecting training data on hardware is costly or risky, a common challenge in robotics. Shih et al. [164] emphasized the role of ML in managing vast datasets used in processing e-skin data for intelligent soft robots. Kim et al. [165] conducted a survey on ML techniques in soft robotics, specifically focusing on feedforward neural networks and recurrent neural networks for control methods, such as proprioception, model-based policy formulation for soft actuator control, and model-free policy formulation. Reinforcement learning techniques are particularly prominent in soft robotics applications, unlike in other fields where alternative methods are more commonly used. Mirza [166] discussed ML’s role in controlling soft robots, whether by directly learning controllers or approximating dynamic models. However, more advancements are required to handle and control soft robots effectively.

Ryan et al. [167] developed a deep learning system for a soft robot using distributed proprioception and soft sensor skin. However, the neural network struggled to accurately predict dynamic motion due to limitations such as the use of voltage dividers and the insufficient current sensitivity of the soft sensor, especially for small dynamic oscillatory motions. Lou et al. [168] proposed a hybrid reinforcement learning control approach for soft robotic arms using hybrid kinematic modeling. They suggest that future research should focus on closed-loop control for precision and dynamic control for rapid applications, leveraging the time-series properties of recurrent neural networks. Huang et al. [169] studied machine learning-based multi-modal information perception for soft robotic hands, concluding that the k-nearest neighbors algorithm combined with double-sensor information outperformed other methods in recognition accuracy, with double-sensor data providing more precise results.

Ding’s study [170] focused on deep learning-based prediction uncertainty for soft robot multimodal sensing, acknowledging that physical changes, such as material stiffening or wear, can reduce prediction model accuracy. Future research could explore how predictive uncertainty could identify delayed distributional shifts and support online learning. Bhagat et al. [171] reviewed DRL mechanisms for soft, flexible robots, exploring the integration of imitation learning and other DRL strategies to create fully autonomous, self-adapting, and physically robust robots capable of replacing humans in various applications.

Thuruthelet al. [172] also reviewed DRL mechanisms for soft, flexible robots, focusing on integrating imitation learning and other DRL techniques to develop fully autonomous, self-adapting soft robots. Wang and Sun [173] evaluated hydrogel-based sensing and actuation techniques in soft robots, predicting that deep learning and physics engines will play an increasingly important role in optimizing soft robotics across various aspects in the future.

In summary, it is clear that ML is driving significant advancements in soft robotics, enhancing control, adaptability, and autonomy.

### 9.3. Internet of Things

The Internet of Things is another key area driving recent developments in soft robotics, revolutionizing industries across the globe. Wang et al. [174] developed a soft robotic platform that integrates Joule heating and IoT technologies. This platform, made from self-prepared boron nitride nanosheets, operates under the cooling towers of thermal power plants, maintaining control in challenging internal conditions and spatial arrangements. Zhang et al. [175] demonstrated that augmented reality and virtual reality can enable designers and users to easily access systems remotely via wireless technology, leading to the realization of the digital twin system. They developed several IoT-based systems, including smart glove-based interfaces, self-powered robotic devices, self-powered socks, and a self-powered smart floor [176]. Sun et al. [176] successfully implemented a digital-twin-based virtual shop using IoT and AI analytics, providing users with real-time feedback on product details. The integration of artificial intelligence of things (AIoT) and ML techniques is transforming certain industries, such as industrial automation, retail, education, and healthcare. These advancements pave the way for the creation of a “smart society,” combining intelligent industrial systems, sensory interactive technologies, and low-cost, high-compatibility solutions [176]. The fusion of AIoT and ML techniques offers a more efficient, cost-effective, and user-friendly approach across various sectors [177]. Borner et al. [178] explored the evolution of AI, robotics, and IoT from 1998 to 2017, noting that the rise of IoT has facilitated robotic applications remotely. Sayeed’s study on the Internet of Robotic Things (IoRT) highlights the sophisticated collaboration between robots and IoT sensors, enhancing IoRT technology and enabling the secure transfer of sensitive data, underlining the potential of these advancements in robotics. Reliable data transfer and sharing systems are crucial for managing security risks, and adopting effective security protocols can ensure secure and efficient data exchange, closing key gaps in the field of IoT-based robotics [177].

Sundaravadivel et al. [179] have enhanced the skill set of the next generation of electrical engineers, preparing them to excel in transdisciplinary applications. Further details on module deployment and evaluations are expected to be gathered in future studies. Yang et al. [180] highlight the significant potential of TENG-enabled wearable sensors and electronics in advancing IoT-integrated green technologies. The development of sustainable IoT systems, characterized by improved wearability, mobility, multifunctionality, low energy consumption, and enhanced intelligence, is poised to continue evolving [181]. AI and ML technologies are revolutionizing event detection by analyzing data from IoT sensor networks, enabling the extraction of crucial information for subsequent actions. This is paving the way for the development of AIoT-integrated green systems [180]. Romeo’s research on IoRT systems suggests that these systems can effectively address the need for remote labor, enhancing productivity and satisfaction through remote human–robot interactions. IoRT systems are expected to contribute significantly to the Fourth Industrial Revolution, necessitating further research and development [181].

Cyber-physical systems and robotic systems are integral to IoRT, requiring additional research to address cyber-physical security challenges. This will enhance the development of smart spaces while addressing concerns regarding the connection between cyber-physical systems and robotic systems [181]. Kua et al. [182] emphasized the critical role of wearable technology in the future of space travel, highlighting its potential for astronauts and space residents. Automation and robotics are anticipated to play a key role in supporting operations, reducing manual labor, and creating new types of robots for specialized applications. Zhang and Ye [183] utilized soft robot technology to compress a human posture recognition model for IoT-enabled human motion tracking. By employing techniques such as Semi-Supervised Learning (SSL) and teacher annealing, they enhanced knowledge distillation, resulting in significant model compression. The model’s accuracy was substantially improved, with the COCO dataset yielding comparable results to HRNet-32. Based on the aforementioned studies, the IoT has paved the way for significant advancements in soft robotics.

### 9.4. Comparative Study of Present Review with Existing Reviews on EAPs for Soft Robotics

Machine learning and the Internet of Things are revolutionizing various sectors of science and technology, including soft robotics. Table 5 presents a comparison between the current review and existing reviews on EAPs and their applications in soft robotics. As shown in Table 5, previous reviews on EAP actuators, artificial muscles, and soft robotics applications have not focused on the role of machine learning and the Internet of Things. In contrast, the emphasis on recent developments in electroactive polymers, along with the integration of machine learning and the Internet of Things in soft robotics, make this review both distinctive and novel compared to previous ones.

## 10. Conclusions and Future Scope

The current work provides a systematic and critical review of EAPs, focusing on applications such as soft robotics and artificial muscle development. Various types of EAPs, including elastomer actuators, LCE actuators, ionic polymer PVC gels, piezoelectric polymers, conducting polymers, IPMCs, are discussed in detail.

Key conclusions drawn from the present study include the following:

1. DEAs can achieve various actuation forms by introducing local stiffness in the elastomer or by rearranging the electrodes.
2. Recent developments in dielectric LCE actuators made from rubber polymers show an exceptional capacity to exert a load 700 times their original weight.
3. Planar PVC gels are capable of stretching up to 600%, offering higher flexibility, reduced weight, and smaller size compared to multilayered PVC gel actuators.
4. Inkjet printing of PVDF in piezoelectric polymers is suitable for developing large-area artificial skin.
5. Conducting polymers are efficient due to their minimal electrolyte content, which allows for sufficient ionic conductivity while maintaining low weight and volume.
6. The interfacial area between the electrode and polymer is crucial for influencing the actuation response and electrochemical behavior in the case of IPMC actuators.
7. MPCs combining magnetic particles with polymer matrices offer a blend of flexibility and magnetic properties. These composites are valuable across industries such as biomedicine, electronics, and environmental engineering thanks to their tunable magnetic, mechanical, and thermal properties.
8. EAPs, ML, and IoT are driving innovations in soft robotics. EAPs are instrumental in creating flexible actuators, while ML and IoT enhance robot control, autonomy, and adaptability.
9. Machine learning techniques, such as reinforcement learning, deep learning, and supervised learning, are transforming soft robotics by optimizing design, control, and sensing. These advancements improve robot functionality, adaptability, and precision.
10. IoT technologies enable advancements in soft robotics by integrating communication systems, remote control, and real-time data feedback. IoT-based platforms, such as smart gloves and self-powered robotic devices, exemplify the evolution of connected soft robotics, with significant implications for industries like automation, healthcare, and retail.

## 11. Future Scope of Soft Robotics

This section discusses future directions for soft robotics, focusing on improved control and self-actuation:

1. Self-healing composites textiles present a promising solution for soft robotics, enabling remote accessibility and drug delivery applications.
2. There is a significant research gap in bio-inspired soft robotics, particularly in optimizing design, manufacturing processes, and control systems.
3. Future research in deep-sea diving robots will focus on integrating low-light imaging, haptics, 3D imaging, microscopy, and genomics to develop tool kits and methodologies for marine biologists.
4. Sustainability in soft robots is a critical concern. Current robots often have negative environmental impacts due to their materials and power sources. Future work should prioritize the development of sustainable alternatives, such as recyclable plastics and biodegradable materials, to minimize environmental damage and promote eco-friendly manufacturing practices.
5. Hydrogels, as smart actuators, can be enhanced using nanomaterials and active stiffness regulation mechanisms. However, their swelling behavior can vary with humidity, causing behavioral abnormalities. To ensure stability, nanoparticles can be integrated to prevent evaporation and enable hydrogels to function as humidity sensors in smart actuators for soft robotics.
6. Systems that incorporate non-electronic information computation in soft robotics are being developed to extract materials and energy from their surroundings. These systems, capable of transforming solar energy into chemical energy for storage and consumption, represent a step towards self-sufficient, self-healing, and potentially self-replicating systems.
7. IoRT systems have the potential to revolutionize industries by addressing remote labor needs and enhancing productivity through human–robot interactions. The continued development of these systems is expected to contribute significantly to the Fourth Industrial Revolution, particularly in sectors that demand advanced robotics.
8. Research can focus on combining different methods, such as blending, in situ polymerization, and molding, for the improved homogeneity and tailored properties of MPCs.
9. Future studies can focus on modifying MPCs to improve their thermal stability and mechanical strength, which are crucial for applications in industries such as aerospace, automotive, and electronics.
10. Eco-friendly synthesis techniques for MPCs should be explored to reduce the use of hazardous solvents and byproducts in production while promoting the development of recyclable or biodegradable materials.

## Figures and Tables

**Table 1:** Potential applications/applied areas of LCE actuators.

Serial Number	Applications/Applied Areas of LCE’s Actuators	Reference
1.	i. irises, ii. biomimetic actuators iii. valves iv. shape-changing lenses, v. reconfigurable topographical surface features to manipulate flow	[19]
2.	i. aerospace ii. robotics iii. medical devices iv. energy harvesting devices v. wearables	[1]

**Table 2:** Potential applications/applied areas of IPMC actuators.

Serial Number	Applications/Applied Areas of IPMC Actuators	Reference
1.	i. active catheters ii. tactile displays iii. braille displays iv. micropumps v. valves vi. robotic grippers vii. sensors	[5]
2.	i. soft robotics ii. artificial muscles iii. biomedical micro-devices	[3]
3.	i. bending actuators	[11]

**Table 3:** Potential applications/applied areas of conducting polymer actuators.

Serial Number	Applications/Applied Areas of Conducting Polymer Actuators	Reference
1.	i. linear/bending actuators ii. origami actuators iii. diaphragm/micro pumps iv. light-emitting diodes v. swimming robots vi. conducting nanowire probes vii drug delivery viii. active catheters ix. batteries x. supercapacitors xi. electrochromic devices xii. selective membranes xiii. braille display xiv. microelectronics xv. sensors	[5]
2.	i. flexible micro-electromechanical systems	[3]
3.	i. steerable catheters ii. micro actuators on optical or electronic circuit boards	[7]

**Table 4:** Comparison of different EAPs based on major operating parameters with key highlights and challenges.

Type of EAPs	Highlights	Challenges	Operating Voltage	Actuation Strain	Response Time	References
Dielectric Elastomers	- Exhibit large strain (up to 300%) - Fast response time - High flexibility	- Requires high voltage - Risk of dielectric breakdown - Requires flexible electrodes	High (in the range of kV)	Up to 300%	Milliseconds	[43,149,150]
Liquid Crystal Elastomers	- Complex deformation can be achieved - Multi-stimuli responsiveness - Exhibit high starin	- Complex fabrication - Limited mechanical strength - Low response time	~100 V	10–300%	Seconds-Minutes	[43,151]
Ionic EAPs	- Operate at low voltage - Flexible and silent operation - Biocompatible	- Requires constant hydration - Low force output - Limited durability	1–5 V	1–10%	Milliseconds-Seconds	[43,152]
Piezoelectric Polymers	- Highly durable - Fast actuation - Ease in fabrication	- Requires high voltage - Actuation strain is less - Environmental senstivity	>0.5–1 kV	0.1–1%	Microseconds-milliseconds	[43,153]
Conducting Polymers	- Electrically conductive - Energy efficient - Tunable properties	- Durability is low - Moisture sensitive - Low force output	1–5 V	2–12%	Seconds-Minutes	[43,92,154]

**Table 5:** Comparison of present review with existing review on EAP’s for soft robotics applications.

Major Theme of Existing Review	Novelty of Present Review with Respect to Existing Review	Difference Between Present and Existing Review/Work	Ref.
Electric Stimulus-Responsive Soft Actuators	machine learning and IoT in robotics	Description of machine learning and IoT are totally absent in this existing review	[145]
Conducting polymers as drug delivery carrier or medium indicated its presence towards application in health-care IoT	recent developments in EAP and machine learning	Description of machine learning is totally absent in this existing review	[184]
Dielectric elastomer actuators for medical applications	recent developments in EAP and machine learning and IoT in robotics	Description of machine learning and IoT are totally absent in this existing review	[185]
Smart polymeric materials	recent developments in EAP and machine learning and IoT in robotics	Description of machine learning and IoT are totally absent in this existing review	[186]
EAP’s for soft robotics and artificial muscle	machine learning and IoT in robotics	Description of machine learning and IoT are totally absent in this existing review	[136]
Bio-inspired soft robotics sensors and actuators	IoT in robotics	Machine learning is partially discussed in existing review but machine learning and IoT both are thoroughly discussed for soft robotics in present review	[187]
Stimuli-Responsive Polymer Actuator for Soft Robotics	machine learning and IoT in robotics	Description of machine learning and IoT are totally absent in this existing review	[188]
Conventional actuators and artificial muscles in upper-limb rehabilitation devices	machine learning and IoT in robotics	Description of machine learning and IoT are totally absent in this existing review	[189]
Ionic Liquid-Based Hybrid Materials for electroactive Soft Actuator Applications	recent developments in EAP and machine learning and IoT in robotics	Description of machine learning and IoT are totally absent in this existing review	[143]
Types and Applications of Soft Robot Arms	machine learning and IoT in robotics	Description of machine learning and IoT are totally absent in this existing review	[190]
Recent advances in soft robotics	machine learning and IoT in robotics	Description of machine learning and IoT are totally absent in this existing review	[3]

## Data Availability

The authors declare that the data supporting the findings of this study are available within the paper.

## References

[1] Qu J., Xu Y., Li Z., Yu Z., Mao B., Wang Y., Wang Z., Fan Q., Qian X., Zhang M. (2024). Recent advances on underwater soft robots. Adv. Intell. Syst..

[2] Rus D., Tolley M.T. (2015). Design, fabrication and control of soft robots. Nature.

[3] Wang Y., Wang Y., Mushtaq R.T., Wei Q. (2024). Advancements in Soft Robotics: A Comprehensive Review on Actuation Methods, Materials, and Applications. Polymers.

[4] AboZaid Y.A., Aboelrayat M.T., Fahim I.S., Radwan A.G. (2024). Soft Robotic Grippers: A Review on Technologies, Materials, and Applications. Sens. Actuators A Phys..

[5] Rodrigue H., Kim J. (2023). Soft actuators in surgical robotics: A state-of-the-art review. Intell. Serv. Robot..

[6] Yao D.R., Kim I., Yin S., Gao W. (2024). Multimodal soft robotic actuation and locomotion. Adv. Mater..

[7] Lee C., Kim M., Kim Y.J., Hong N., Ryu S., Kim H.J., Kim S. (2017). Soft robot review. Int. J. Control. Autom. Syst..

[8] Wang J., Chortos A. (2022). Control strategies for soft robot systems. Adv. Intell. Syst..

[9] Miriyev A., Stack K., Lipson H. (2017). Soft material for soft actuators. Nat. Commun..

[10] Kim J., Kim J.W., Kim H.C., Zhai L., Ko H.U., Muthoka R.M. (2019). Review of soft actuator materials. Int. J. Precis. Eng. Manuf..

[11] Elango N., Faudzi A.A.M. (2015). A review article: Investigations on soft materials for soft robot manipulations. Int. J. Adv. Manuf. Technol..

[12] Yang Y., Wu Y., Li C., Yang X., Chen W. (2020). Flexible actuators for soft robotics. Adv. Intell. Syst..

[13] Bar-Cohen Y. (2002). Electroactive polymers as artificial muscles: A review. J. Spacecr. Rocket..

[14] Enyan M., Bing Z., Amu-Darko J.N.O., Issaka E., Otoo S.L., Agyemang M.F. (2024). Advances in smart materials soft actuators on mechanisms, fabrication, materials, and multifaceted applications: A review. J. Thermoplast. Compos. Mater..

[15] Wang Z., Chen Y., Ma Y., Wang J. (2024). Bioinspired Stimuli-Responsive Materials for Soft Actuators. Biomimetics.

[16] Guo Y., Wang Y., Tong Q., Shan B., He L., Zhang Y., Wang D. (2024). Active electronic skin: An interface towards ambient haptic feedback on physical surfaces. Npj Flex. Electron..

[17] White B.T., Long T.E. (2019). Advances in polymeric materials for electromechanical devices. Macromol. Rapid Commun..

[18] Cianchetti M., Laschi C., Menciassi A., Dario P. (2018). Biomedical applications of soft robotics. Nat. Rev. Mater..

[19] El-Atab N., Mishra R.B., Al-Modaf F., Joharji L., Alsharif A.A., Alamoudi H., Diaz M., Qaiser N., Hussain M.M. (2020). Soft actuators for soft robotic applications: A review. Adv. Intell. Syst..

[20] Zhang Y., Li P., Quan J., Li L., Zhang G., Zhou D. (2023). Progress, challenges, and prospects of soft robotics for space applications. Adv. Intell. Syst..

[21] Dong X., Luo X., Zhao H., Qiao C., Li J., Yi J., Yang L., Oropeza F.J., Hu T.S., Xu Q. (2022). Recent advances in biomimetic soft robotics: Fabrication approaches, driven strategies and applications. Soft Matter.

[22] Greco C., Kotak P., Pagnotta L., Lamuta C. (2022). The evolution of mechanical actuation: From conventional actuators to artificial muscles. Int. Mater. Rev..

[23] Lin W., Wei Y., Wang X., Zhai K., Ji X. (2023). Study on Human Motion Energy Harvesting Devices: A Review. Machines.

[24] Gu G.-Y., Zhu J., Zhu L.-M., Zhu X. (2017). A survey on dielectric elastomer actuators for soft robots. Bioinspiration Biomim..

[25] Mutlu R., Alici G., Xiang X., Li W. (2014). Electro-mechanical modelling and identification of electroactive polymer actuators as smart robotic manipulators. Mechatronics.

[26] Zhao W., Zhang Y., Wang N. (2021). Soft robotics: Research, challenges, and prospects. J. Robot. Mechatron..

[27] Apsite I., Salehi S., Ionov L. (2021). Materials for smart soft actuator systems. Chem. Rev..

[28] Guin T., Settle M.J., Kowalski B.A., Auguste A.D., Beblo R.V., Reich G.W., White T.J. (2018). Layered liquid crystal elastomer actuators. Nat. Commun..

[29] Shahinpoor M. Electrically activated artificial muscles made with liquid crystal elastomers. Proceedings of the SPIE’s 7th Annual International Symposium on Smart Structures and Materials.

[30] Madden J.D.W. (2000). Conducting Polymer Actuators. Ph.D. Dissertation.

[31] Finkelmann H., Shahinpoor M. Electrically controllable liquid crystal elastomer-graphite composite artifical muscles. Proceedings of the SPIE’s 9th Annual International Symposium on Smart Structures and Materials.

[32] Davidson Z.S., Shahsavan H., Aghakhani A., Guo Y., Hines L., Xia Y., Yang S., Sitti M. (2019). Monolithic shape-programmable dielectric liquid crystal elastomer actuators. Sci. Adv..

[33] Bar-Cohen Y., Zhang Q. (2008). Electroactive polymer actuators and sensors. MRS Bull..

[34] Carpi F., Kornbluh R., Sommer-Larsen P., Alici G. (2011). Electroactive polymer actuators as artificial muscles: Are they ready for bioinspired applications?. Bioinspir. Biomim..

[35] Marín F., Martínez-Frutos J., Ortigosa R., Gil A. (2021). A convex multi-variable based computational framework for multilayered electro-active polymers. Comput. Methods Appl. Mech. Eng..

[36] Kallitsis K., Thuau D., Brochon C., Cloutet E., Hadziioannou G. (2021). Tailoring fluorinated electroactive polymers toward specific applications. Colloid Polym. Sci..

[37] Xia F., Tadigadapa S., Zhang Q. (2005). Electroactive polymer based microfluidic pump. Sensors Actuators A Phys..

[38] Hartmann F., Penkner L., Danninger D., Arnold N., Kaltenbrunner M. (2021). Soft tunable lenses based on zipping electroactive polymer actuators. Adv. Sci..

[39] Jo A., Huet C., Naguib H.E. (2020). Template-assisted self-assembly of conductive polymer electrodes for ionic electroactive polymers. Front. Bioeng. Biotechnol..

[40] Kim O., Kim S.J., Park M.J. (2018). Low-voltage-driven soft actuators. Chem. Commun..

[41] Baughman R.H. (1996). Conducting polymer artificial muscles. Synth. Met..

[42] Rahman M.H., Werth H., Goldman A., Hida Y., Diesner C., Lane L., Menezes P.L. (2021). Recent progress on electroactive polymers: Synthesis, properties and applications. Ceramics.

[43] Maksimkin A.V., Dayyoub T., Telyshev D.V., Gerasimenko A.Y. (2022). Electroactive polymer-based composites for artificial muscle-like actuators: A review. Nanomaterials.

[44] Bashir M., Rajendran P. (2018). A review on electroactive polymers development for aerospace applications. J. Intell. Mater. Syst. Struct..

[45] Guo Y., Liu L., Liu Y., Leng J. (2021). Review of dielectric elastomer actuators and their applications in soft robots. Adv. Intell. Syst..

[46] Qiu Y., Zhang E., Plamthottam R., Pei Q. (2019). Dielectric elastomer artificial muscle: Materials innovations and device explorations. Accounts Chem. Res..

[47] Liu Z., Liu Y.D., Shi Q., Liang Y. (2021). Electroactive dielectric polymer gels as new-generation soft actuators: A review. J. Mater. Sci..

[48] Shankar R., Ghosh T.K., Spontak R.J. (2007). Dielectric elastomers as next-generation polymeric actuators. Soft Matter.

[49] Liu L., Zhang J., Luo M., Chen H., Yang Z., Li D., Li P. (2020). A bio-inspired soft-rigid hybrid actuator made of electroactive dielectric elastomers. Appl. Mater. Today.

[50] Wang J., Gao D., Lee P.S. (2021). Recent progress in artificial muscles for interactive soft robotics. Adv. Mater..

[51] Wang Y., Ma X., Jiang Y., Zang W., Cao P., Tian M., Ning N., Zhang L. (2022). Dielectric elastomer actuators for artificial muscles: A comprehensive review of soft robot explorations. Resour. Chem. Mater..

[52] Li J., Liu L., Liu Y., Leng J. (2019). Dielectric elastomer spring-roll bending actuators: Applications in soft robotics and design. Soft Robot..

[53] Adachi M., Hamazawa K., Mimuro Y. (2017). Vibration transport system for lunar and Martian regolith using dielectric elastomer actuator. J. Electrost..

[54] Chen F., Wang M.Y., Zhu J., Zhang Y.F. (2016). Interactions between dielectric elastomer actuators and soft bodies. Soft Robot..

[55] Thongking W., Wiranata A., Minaminosono A., Mao Z., Maeda S. (2021). Soft robotic gripper based on multi-layers of dielectric elastomer actuators. J. Robot. Mechatronics.

[56] Kofod G., Wirges W., Paajanen M., Bauer S. (2007). Energy minimization for self-organized structure formation and actuation. Appl. Phys. Lett..

[57] Schadt M. (1997). Liquid crystal materials and liquid crystal displays. Annu. Rev. Mater. Sci..

[58] Marrucci G., Greco F. (1993). Flow behavior of liquid crystalline polymers. Adv. Chem. Phys..

[59] Cao S., Aimi J., Yoshio M. (2022). Electroactive soft actuators based on columnar ionic liquid crystal/polymer composite membrane electrolytes forming 3D continuous ionic channels. ACS Appl. Mater. Interfaces.

[60] Liu G., Deng Y., Ni B., Nguyen G.T.M., Vancaeyzeele C., Brûlet A., Vidal F., Plesse C., Li M. (2024). Electroactive Bi-Functional Liquid Crystal Elastomer Actuators. Small.

[61] Xiao Y.Y., Jiang Z.C., Zhao Y. (2020). Liquid crystal polymer-based soft robots. Adv. Intell. Syst..

[62] Lehmann W., Skupin H., Tolksdorf C., Gebhard E., Zentel R., Krüger P., Lösche M., Kremer F. (2001). Giant lateral electrostriction in ferroelectric liquid-crystalline elastomers. Nature.

[63] Madden J., Vandesteeg N., Anquetil P., Madden P., Takshi A., Pytel R., Lafontaine S., Wieringa P., Hunter I. (2004). Artificial muscle technology: Physical principles and naval prospects. IEEE J. Ocean. Eng..

[64] Palffy-Muhoray P. (2009). Printed actuators in a flap. Nat. Mater..

[65] Nemat-Nasser S., Wu Y. (2003). Comparative experimental study of ionic polymer–metal composites with different backbone ionomers and in various cation forms. J. Appl. Phys..

[66] Noh T.-G., Tak Y., Nam J.-D., Choi H. (2002). Electrochemical characterization of polymer actuator with large interfacial area. Electrochimica Acta.

[67] Kim K.J., Shahinpoor M. (2003). Ionic polymer–metal composites: II. Manufacturing techniques. Smart Mater. Struct..

[68] Luqman M., Anis A., Shaikh H.M., Al-Zahrani S.M., Alam M.A. (2022). Development of a Soft Robotic Bending Actuator Based on a Novel Sulfonated Polyvinyl Chloride–Phosphotungstic Acid Ionic Polymer–Metal Composite (IPMC) Membrane. Membranes.

[69] Mirvakili S.M., Hunter I.W. (2018). Artificial muscles: Mechanisms, applications, and challenges. Adv. Mater..

[70] Ahn K.K., Truong D.Q., Nam D.N.C., Yoon J.I., Yokota S. (2010). Position control of ionic polymer metal composite actuator using quantitative feedback theory. Sens. Actuators A Phys..

[71] Li Y., Hashimoto M. (2015). PVC gel based artificial muscles: Characterizations and actuation modular constructions. Sens. Actuators A Phys..

[72] Xia H., Takasaki M., Hirai T. (2010). Actuation mechanism of plasticized PVC by electric field. Sens. Actuators A Phys..

[73] Basinski Z.S., Christian J. (1954). Crystallography of deformation by twin boundary movements in indium-thallium alloys. Acta Met..

[74] Li Y., Li Y., Hashimoto M. (2019). Low-voltage planar PVC gel actuator with high performances. Sens. Actuators B Chem..

[75] Sharif M.A. (2022). PVC gel smart sensor for robotics sensing applications: An experimental and finite element simulation study. Eng. Res. Express.

[76] Xie M., Hisano K., Zhu M., Toyoshi T., Pan M., Okada S., Tsutsumi O., Kawamura S., Bowen C. (2019). Flexible multifunctional sensors for wearable and robotic applications. Adv. Mater. Technol..

[77] Sohn J.W., Choi S.B. (2017). Various robots made from piezoelectric materials and electroactive polymers: A review. Int. J. Mech. Syst. Eng..

[78] Guo J., Nie M., Wang Q. (2021). A piezoelectric poly (vinylidene fluoride) tube featuring highly-sensitive and isotropic piezoelectric output for compression. RSC Adv..

[79] Xin Y., Tian H., Guo C., Li X., Sun H., Wang P., Lin J., Wang S., Wang C. (2016). PVDF tactile sensors for detecting contact force and slip: A review. Ferroelectrics.

[80] Mishra S., Unnikrishnan L., Nayak S.K., Mohanty S. (2018). Advances in piezoelectric polymer composites for energy harvesting applications: A systematic review. Macromol. Mater. Eng..

[81] Kim D.H., Kim B., Kang H. (2004). Development of a piezoelectric polymer-based sensorized microgripper for microassembly and micromanipulation. Microsyst. Technol..

[82] Tian H. (2018). A robot attached the soft sensor using PVDF film for objects discrimination. Integr. Ferroelectr..

[83] Seminara L., Pinna L., Valle M., Basirico L., Loi A., Cosseddu P., Bonfiglio A., Ascia A., Biso M., Ansaldo A. (2013). Piezoelectric polymer transducer arrays for flexible tactile sensors. IEEE Sens. J..

[84] Kimoto A., Sugitani N., Fujisaki S. (2010). A multifunctional tactile sensor based on PVDF films for identification of materials. IEEE Sens. J..

[85] Hosoda K., Tada Y., Asada M. (2006). Anthropomorphic robotic soft fingertip with randomly distributed receptors. Robot. Auton. Syst..

[86] Namvarrechi S. (2021). Fabrication, Characterization and Modelling of Piezoelectric PVDF-TrFE Polymer as a Force Sensor Using Spin Coating Method. Ph.D. Dissertation.

[87] Gupta S., Shakthivel D., Lorenzelli L., Dahiya R. (2018). Temperature compensated tactile sensing using MOSFET with P (VDF-TrFE)/BaTiO_3_ capacitor as extended gate. IEEE Sens. J..

[88] Smith M., Kar-Narayan S. (2022). Piezoelectric polymers: Theory, challenges and opportunities. Int. Mater. Rev..

[89] Melling D., Martinez J.G., Jager E.W.H. (2019). Conjugated polymer actuators and devices: Progress and opportunities. Adv. Mater..

[90] Das T.K., Prusty S. (2012). Review on conducting polymers and their applications. Polym. Technol. Eng..

[91] Martins P., Correia D.M., Correia V., Lanceros-Mendez S. (2020). Polymer-based actuators: Back to the future. Phys. Chem. Chem. Phys..

[92] Otero T.F. (2013). Biomimetic conducting polymers: Synthesis, materials, properties, functions, and devices. Polym. Rev..

[93] Namsheer K., Rout C.S. (2021). Conducting polymers: A comprehensive review on recent advances in synthesis, properties and applications. RSC Adv..

[94] Sharma S., Sudhakara P., Omran A.A.B., Singh J., Ilyas R.A. (2021). Recent trends and developments in conducting polymer nanocomposites for multifunctional applications. Polymers.

[95] Ma Z., Shi W., Yan K., Pan L., Yu G. (2019). Doping engineering of conductive polymer hydrogels and their application in advanced sensor technologies. Chem. Sci..

[96] Lu Y., Wang J.-Y., Pei J. (2021). Achieving efficient n-doping of conjugated polymers by molecular dopants. Acc. Chem. Res..

[97] Jacobs I.E., Lin Y., Huang Y., Ren X., Simatos D., Chen C., Tjhe D., Statz M., Lai L., Finn P.A. (2022). High-Efficiency Ion-Exchange Doping of Conducting Polymers. Adv. Mater..

[98] Ma S., Xue P., Valenzuela C., Zhang X., Chen Y., Liu Y., Yang L., Xu X., Wang L. (2024). Highly Stretchable and Conductive MXene-Encapsulated Liquid Metal Hydrogels for Bioinspired Self-Sensing Soft Actuators. Adv. Funct. Mater..

[99] Hu H., Zhang S., Xu J., Salim T., Li Y., Hu X., Zhang Z., Cheng G., Yuan N., Lam Y.M. (2024). Thermal-sensing actuator based on conductive polymer ionogel for autonomous human-machine interaction. Sens. Actuators B Truongchem..

[100] Liu L., Li Y., Lu Z., Miao R., Zhang N. (2024). Thermal and light-driven soft actuators based on a conductive polypyrrole nanofibers integrated poly (N-isopropylacrylamide) hydrogel with intelligent response. J. Colloid Interface Sci..

[101] Hu H., Zhang S., Zhang M., Xu J., Salim T., Li Y., Hu X., Zhang Z., Cheng G., Yuan N. (2024). Artificial Muscles Based on Coiled Conductive Polymer Yarns. Adv. Funct. Mater..

[102] Périgo E.A., Weidenfeller B., Kollár P., Füzer J. (2018). Past, present, and future of soft magnetic composites. Appl. Phys. Rev..

[103] Parveen S., Misra R., Sahoo S.K. (2012). Nanoparticles: A boon to drug delivery, therapeutics, diagnostics and imaging. Nanomed. Nanotechnol. Biol. Med..

[104] Shubayev V.I., Pisanic T.R., Jin S. (2009). Magnetic nanoparticles for theragnostics. Adv. Drug Deliv. Rev..

[105] Cole A.J., Yang V.C., David A.E. (2011). Cancer theranostics: The rise of targeted magnetic nanoparticles. Trends Biotechnol..

[106] Sharma R., Sharma A., Chen C. (2011). State of art on bioimaging by nanoparticles in hyperthermia and thermometry: Visualization of tissue protein targeting. Open Nanomed. J..

[107] Audonnet V., Malaquin L., Viovy J.-L. (2011). Polymeric coatings on micro- and nanometric particles for bioapplications. Bioanal. Rev..

[108] Gijs M.A.M., Lacharme F., Lehmann U. (2009). Microfluidic Applications of Magnetic Particles for Biological Analysis and Catalysis. Chem. Rev..

[109] Lu Q., Choi K., Nam J.-D., Choi H.J. (2021). Magnetic Polymer Composite Particles: Design and Magnetorheology. Polymers.

[110] Keçili R., Büyüktiryaki S., Dolak I., Hussain C.M. (2020). The use of magnetic nanoparticles in sample preparation devices and tools. Handbook of Nanomaterials in Analytical Chemistry.

[111] Haldorai Y., Shim J.-J., Lim K.T. (2012). Synthesis of polymer–inorganic filler nanocomposites in supercritical CO_2_. J. Supercrit. Fluids.

[112] Chung Y.-C., Choi J.W., Choi M.W., Chun B.C. (2012). Characterization of flexibly linked shape memory polyurethane composite with magnetic property. J. Thermoplast. Compos. Mater..

[113] Rajput A.B., Rahaman S.J., Sarkhel G., Patra M.K., Vadera S.R., Singru P.M., Yagci Y., Ghosh N. (2012). Synthesis, characterization, and properties of flexible magnetic nanocomposites of cobalt ferrite–polybenzoxazine–linear low-density polyethylene. J. Appl. Polym. Sci..

[114] Noor A., Ibreheim H.S. (2019). Effect of ZrO_2_ on Mechanical Strength, Antibacterial, and Anti Fungal of Epoxy Adhesive. Indian J. Nat. Sci..

[115] Arias A., Heuzey M.-C., Huneault M.A., Ausias G., Bendahou A. (2015). Enhanced dispersion of cellulose nanocrystals in melt-processed polylactide-based nanocomposites. Cellulose.

[116] Cherifi Z., Zaoui A., Boukoussa B., Derdar H., El Abed O.Z., Zeggai F.Z., Meghabar R., Chebout R. (2022). Ultrasound-promoted preparation of cellulose acetate/organophilic clay bio-nanocomposites films by solvent casting method. Polym. Bull..

[117] Bustamante-Torres M., Romero-Fierro D., Estrella-Nuñez J., Arcentales-Vera B., Chichande-Proaño E., Bucio E. (2022). Polymeric Composite of Magnetite Iron Oxide Nanoparticles and Their Application in Biomedicine: A Review. Polymers.

[118] Soykan C., Akbay M. (2019). In-Situ synthesis of polymer–clay nanocomposites: Exfoliation of organophilic montmorillonite nanolayers in poly 2-thiozylmethacrylamide. J. Mater. Sci. Technol. Res..

[119] Guo F., Aryana S., Han Y., Jiao Y. (2018). A Review of the Synthesis and Applications of Polymer–Nanoclay Composites. Appl. Sci..

[120] Shabzendedar S., Modarresi-Alam A.R., Noroozifar M., Kerman K. (2020). Core-shell nanocomposite of superparamagnetic Fe_3_O_4_ nanoparticles with poly(m-aminobenzenesulfonic acid) for polymer solar cells. Org. Electron..

[121] Wang Y., Zhu Y., Xue Y., Wang J., Li X., Wu X., Qin Y., Chen W. (2020). Sequential in-situ route to synthesize novel composite hydrogels with excellent mechanical, conductive, and magnetic responsive properties. Mater. Des..

[122] Romero-Fierro D., Bustamante-Torres M., Bravo-Plascencia F., Magaña H., Bucio E. (2022). Polymer-Magnetic Semiconductor Nanocomposites for Industrial Electronic Applications. Polymers.

[123] Chen A., Wang Q., Li M., Peng Z., Lai J., Zhang J., Xu J., Huang H. (2021). Combined approach of compression molding and magnetic attraction to micropatterning of magnetic polydimethylsiloxane composite surfaces with excellent anti-icing/deicing performance. ACS Appl. Mater. Interfaces.

[124] Gautam R.K., Chattopadhyaya M.C. (2016). Functionalized Magnetic Nanoparticles: Adsorbents and Applications. Nanomaterials for Wastewater Remediation.

[125] Jiang Y., Liang F., Li H.Y., Li X., Fan Y.J., Cao J.W., Yin Y.M., Wang Y., Wang Z.L., Zhu G. (2022). A flexible and ultra-highly sensitive tactile sensor through a parallel circuit by a magnetic aligned conductive composite. ACS Nano.

[126] Jiles D., Lo C. (2003). The role of new materials in the development of magnetic sensors and actuators. Sens. Actuators A Phys..

[127] Varga Z., Filipcsei G., Zrínyi M. (2006). Magnetic field sensitive functional elastomers with tuneable elastic modulus. Polymer.

[128] Urso M., Ussia M., Novotný F., Pumera M. (2022). Trapping and detecting nanoplastics by MXene-derived oxide microrobots. Nat. Commun..

[129] Mamatha G.M., Pradipkumar D.R., Hari K., Girish K.S. (2024). Polymer based composites for electromagnetic interference (EMI) shielding: The role of magnetic fillers in effective attenuation of microwaves, a review. Hybrid Adv..

[130] Ganguly S., Margel S. (2023). Fabrication and Applications of Magnetic Polymer Composites for Soft Robotics. Micromachines.

[131] Ganguly S., Margel S. (2025). Magnetic Polymeric Conduits in Biomedical Applications. Micromachines.

[132] Sasmal A., Sen S., Arout Chelvane J., Arockiarajan A. (2023). PVDF based flexible magnetoelectric composites for capacitive energy storage, hybrid mechanical energy harvesting and self-powered magnetic field detection. Polymer.

[133] Lu F., Chen T., Xiang K., Wang Y. (2020). Ionic electro-active polymer actuator based on cobalt-containing nitrogen-doped carbon/conducting polymer soft electrode. Polym. Test..

[134] Khalid M.Y., Arif Z.U., Tariq A., Hossain M., Khan K.A., Umer R. (2024). 3D printing of magneto-active smart materials for advanced actuators and soft robotics applications. Eur. Polym. J..

[135] Hassan H., Hallez H., Thielemans W., Vandeginste V. (2024). A review of electro-active shape memory polymer composites: Materials engineering strategies for shape memory enhancement. Eur. Polym. J..

[136] Yang L., Wang H. (2024). High-performance electrically responsive artificial muscle materials for soft robot actuation. Acta Biomater..

[137] Reghunadhan A., Krishna A., Jose A.J. (2020). Polymers in robotics. Polymer Science and Innovative Applications.

[138] Trümpler N., Kanno R., David N., Huch A., Nguyen P.H., Jurinovs M., Nyström G., Gaidukovs S., Kovac M. (2025). Low-Cost 3D printed, Biocompatible Ionic Polymer Membranes for Soft Actuators. arXiv.

[139] Jumet B., Bell M.D., Sanchez V., Preston D.J. (2020). A data-driven review of soft robotics. Adv. Intell. Syst..

[140] Chen Y., Zhang F., Cheng G., Jiao J., Zhang Z. (2021). Advances in electroactive polymer-based haptic actuators for human-machine interfaces: From principles to applications. Nanomaterials.

[141] Bernat J., Gajewski P., Kołota J., Marcinkowska A. (2023). Review of soft actuators controlled with electrical stimuli: IPMC, DEAP, and MRE. Appl. Sci..

[142] Biswal D.K. (2023). Application of Electroactive Polymer Actuator: A Brief Review. Biomimicry Materials and Applications.

[143] Fernandes L., Correia D., Costa C., Lanceros-Mendez S. (2024). Recent Advances in Ionic Liquid-Based Hybrid Materials for Electroactive Soft Actuator Applications. Macromol. Mater. Eng..

[144] Kanaan A.F., Pinho A.C., Piedade A.P. (2021). Electroactive polymers obtained by conventional and non-conventional technologies. Polymers.

[145] Jo S.J., Kim G.M., Kim J. (2024). Recent Advances in Electric Stimulus-Responsive Soft Actuators. Compos. Res..

[146] Deng C., Li Z. (2025). Advanced Drive Technologies for Bionic Soft Robots. J. Bionic Eng..

[147] Beregoi M., Beaumont S., Evanghelidis A., Otero T.F., Enculescu I. (2022). Bioinspired polypyrrole based fibrillary artificial muscle with actuation and intrinsic sensing capabilities. Sci. Rep..

[148] Bruns M., Mehraeen S., Martinez J.G., Cherif C., Jager E.W.H. (2025). PEDOT/Polypyrrole Core–Sheath Fibers for Use as Conducting Polymer Artificial Muscles. ACS Appl. Mater. Interfaces.

[149] Brochu P., Pei Q. (2012). Dielectric elastomers for actuators and artificial muscles. Electroactivity in Polymeric Materials.

[150] Guo Y., Qin Q., Han Z., Plamthottam R., Possinger M., Pei Q. (2023). Dielectric elastomer artificial muscle materials advancement and soft robotic applications. SmartMat.

[151] Jiang H., Li C., Huang X. (2013). Actuators based on liquid crystalline elastomer materials. Nanoscale.

[152] Shahinpoor M., Bar-Cohen Y., Simpson J.O., Smith J. (1998). Ionic polymer-metal composites (IPMCs) as biomimetic sensors, actuators and artificial muscles-a review. Smart Mater. Struct..

[153] Ariano P., Accardo D., Lombardi M., Bocchini S., Draghi L., De Nardo L., Fino P. (2015). Polymeric materials as artificial muscles: An overview. J. Appl. Biomater. Funct. Mater..

[154] Le T.-H., Kim Y., Yoon H. (2017). Electrical and electrochemical properties of conducting polymers. Polymers.

[155] Yin S., Jia Z., Li X., Zhu J., Xu Y., Li T. (2022). Machine-learning-accelerated design of functional structural components in deep-sea soft robots. Extreme Mech. Lett..

[156] Chin K., Hellebrekers T., Majidi C. (2020). Machine learning for soft robotic sensing and control. Adv. Intell. Syst..

[157] Yao J., Cao Q., Ju Y., Sun Y., Liu R., Han X., Li L. (2023). Adaptive actuation of magnetic soft robots using deep reinforcement learning. Adv. Intell. Syst..

[158] Čakurda T., Trojanová M., Pomin P., Hošovský A. (2024). Deep Learning Methods in Soft Robotics: Architectures and Applications. Adv. Intell. Syst..

[159] Yasa O., Toshimitsu Y., Michelis M.Y., Jones L.S., Filippi M., Buchner T., Katzschmann R.K. (2023). An overview of soft robotics. Annu. Rev. Control. Robot. Auton. Syst..

[160] Terrile S., López A., Barrientos A. (2023). Use of Finite Elements in the Training of a Neural Network for the Modeling of a Soft Robot. Biomimetics.

[161] Tsompanas M.-A., You J., Philamore H., Rossiter J., Ieropoulos I. (2021). Neural networks predicting microbial fuel cells output for soft robotics applications. Front. Robot. AI.

[162] Raeisinezhad M., Pagliocca N., Koohbor B., Trkov M. (2021). Design optimization of a pneumatic soft robotic actuator using model-based optimization and deep reinforcement learning. Front. Robot. AI.

[163] Johnson C.C., Quackenbush T., Sorensen T., Wingate D., Killpack M.D. (2021). Using first principles for deep learning and model-based control of soft robots. Front. Robot. AI.

[164] Shih B., Shah D., Li J., Thuruthel T.G., Park Y.-L., Iida F., Bao Z., Kramer-Bottiglio R., Tolley M.T. (2020). Electronic skins and machine learning for intelligent soft robots. Sci. Robot..

[165] Kim D., Kim S.H., Kim T., Kang B.B., Lee M., Park W., Ku S., Kim D., Kwon J., Lee H. (2021). Review of machine learning methods in soft robotics. PLoS ONE.

[166] Mirza N.M. Machine learning and soft robotics. Proceedings of the 2020 21st International Arab Conference on Information Technology (ACIT).

[167] Truby R.L., Della Santina C., Rus D. (2020). Distributed proprioception of 3D configuration in soft, sensorized robots via deep learning. IEEE Robot. Autom. Lett..

[168] Lou G., Wang C., Xu Z., Liang J., Zhou Y. (2024). Controlling soft robotic arms using hybrid modelling and reinforcement learning. IEEE Robot. Autom. Lett..

[169] Huang H., Lin J., Wu L., Fang B., Wen Z., Sun F. (2020). Machine learning-based multi-modal information perception for soft robotic hands. Tsinghua Sci. Technol..

[170] Ding Z.Y., Loo J.Y., Baskaran V.M., Nurzaman S.G., Tan C.P. (2021). Predictive uncertainty estimation using deep learning for soft robot multimodal sensing. IEEE Robot. Autom. Lett..

[171] Bhagat S., Banerjee H., Ho Tse Z.T., Ren H. (2019). Deep reinforcement learning for soft, flexible robots: Brief review with impending challenges. Robotics.

[172] Thuruthel T.G., Falotico E., Renda F., Laschi C. (2018). Model-based reinforcement learning for closed-loop dynamic control of soft robotic manipulators. IEEE Trans. Robot..

[173] Wang S., Sun Z. (2023). Hydrogel and machine learning for soft robots’ sensing and signal processing: A review. J. Bionic Eng..

[174] Wang L., Sun X., Wang D., Cui P., Wang J., Li Q. (2024). High-precision, programmable soft wireless robotics for cooling tower cleaning based on Internet of Things technology. Chem. Eng. J..

[175] Zhang Z., Wen F., Sun Z., Guo X., He T., Lee C. (2022). Artificial intelligence-enabled sensing technologies in the 5G/internet of things era: From virtual reality/augmented reality to the digital twin. Adv. Intell. Syst..

[176] Sun Z., Zhu M., Zhang Z., Chen Z., Shi Q., Shan X., Yeow R.C.H., Lee C. (2021). Artificial Intelligence of Things (AIoT) enabled virtual shop applications using self-powered sensor enhanced soft robotic manipulator. Adv. Sci..

[177] Sayeed A., Verma C., Kumar N., Koul N., Illés Z. (2022). Approaches and challenges in Internet of robotic things. Future Internet.

[178] Börner K., Scrivner O., Cross L.E., Gallant M., Ma S., Martin A.S., Record L., Yang H., Dilger J.M. (2020). Mapping the co-evolution of artificial intelligence, robotics, and the internet of things over 20 years (1998–2017). PLoS ONE.

[179] Sundaravadivel P., Ghosh P.K., Suwal B. IoT-enabled Soft Robotics for Electrical Engineers. Proceedings of the Great Lakes Symposium on VLSI 2022.

[180] Yang Y., Guo X., Zhu M., Sun Z., Zhang Z., He T., Lee C. (2022). Triboelectric nanogenerator enabled wearable sensors and electronics for sustainable internet of things integrated green earth. Adv. Energy Mater..

[181] Romeo L., Petitti A., Marani R., Milella A. (2020). Internet of robotic things in smart domains: Applications and challenges. Sensors.

[182] Kua J., Loke S.W., Arora C., Fernando N., Ranaweera C. (2021). Internet of things in space: A review of opportunities and challenges from satellite-aided computing to digitally-enhanced space living. Sensors.

[183] Ye L., Zheng Y. (2022). The Image Processing Using Soft Robot Technology in Fitness Motion Detection Under the Internet of Things. IEEE Access.

[184] Alkahtani M.E., Elbadawi M., Chapman C.A., Green R.A., Gaisford S., Orlu M., Basit A.W. (2024). Electroactive Polymers for On-Demand Drug Release. Adv. Healthc. Mater..

[185] Ghevondyan M., Davtyan M., Aghayan M. (2025). Dielectric elastomer actuators: Medical applications review. Discov. Mater..

[186] Fattah-Alhosseini A., Chaharmahali R., Alizad S., Kaseem M., Dikici B. (2024). A review of smart polymeric materials: Recent developments and prospects for medicine applications. Hybrid Adv..

[187] Sarker A., Islam T.U., Islam R. (2024). A Review on Recent Trends of Bioinspired Soft Robotics: Actuators, Control Methods, Materials Selection, Sensors, Challenges, and Future Prospects. Adv. Intell. Syst..

[188] Kim S., Lee S.-N., Melvin A.A., Choi J.-W. (2024). Stimuli-Responsive Polymer Actuator for Soft Robotics. Polymers.

[189] Garofalo S., Morano C., Perrelli M., Pagnotta L., Carbone G., Mundo D., Bruno L. (2024). A critical review of transitioning from conventional actuators to artificial muscles in upper-limb rehabilitation devices. J. Intell. Mater. Syst. Struct..

[190] Mohammed M., Al-Ibadi A. (2024). Types and Applications of Soft Robot Arms and End-Effectors: A Review. J. Robot. Res..

